# Noncanonical and reversible cysteine ubiquitination prevents the overubiquitination of PEX5 at the peroxisomal membrane

**DOI:** 10.1371/journal.pbio.3002567

**Published:** 2024-03-12

**Authors:** Tânia Francisco, Ana G. Pedrosa, Tony A. Rodrigues, Tarad Abalkhail, Hongli Li, Maria J. Ferreira, Gerbrand J. van der Heden van Noort, Marc Fransen, Ewald H. Hettema, Jorge E. Azevedo

**Affiliations:** 1 Instituto de Investigação e Inovação em Saúde (i3S), Universidade do Porto, Porto, Portugal; 2 Instituto de Biologia Molecular e Celular (IBMC), Universidade do Porto, Porto, Portugal; 3 Instituto de Ciências Biomédicas Abel Salazar (ICBAS), Universidade do Porto, Porto, Portugal; 4 School of Biosciences, University of Sheffield, Sheffield, United Kingdom; 5 Laboratory of Peroxisome Biology and Intracellular Communication, Department of Cellular and Molecular Medicine, Katholieke Universiteit Leuven, Leuven, Belgium; 6 Department of Cell and Chemical Biology, Leiden University Medical Center, Leiden, the Netherlands; Princeton University, UNITED STATES

## Abstract

PEX5, the peroxisomal protein shuttling receptor, binds newly synthesized proteins in the cytosol and transports them to the organelle. During its stay at the peroxisomal protein translocon, PEX5 is monoubiquitinated at its cysteine 11 residue, a mandatory modification for its subsequent ATP-dependent extraction back into the cytosol. The reason why a cysteine and not a lysine residue is the ubiquitin acceptor is unknown. Using an established rat liver-based cell-free in vitro system, we found that, in contrast to wild-type PEX5, a PEX5 protein possessing a lysine at position 11 is polyubiquitinated at the peroxisomal membrane, a modification that negatively interferes with the extraction process. Wild-type PEX5 cannot retain a polyubiquitin chain because ubiquitination at cysteine 11 is a reversible reaction, with the E2-mediated deubiquitination step presenting faster kinetics than PEX5 polyubiquitination. We propose that the reversible nonconventional ubiquitination of PEX5 ensures that neither the peroxisomal protein translocon becomes obstructed with polyubiquitinated PEX5 nor is PEX5 targeted for proteasomal degradation.

## Abstract 2

The peroxisomal protein shuttling receptor PEX5 transports newly synthesised proteins from the cytosol to the peroxisome, where it is monoubiquitinated at cysteine 11 so it can be extracted back to the cytosol by ATP. These authors show that longer ubiquitin chains attached to this cysteine are rapidly removed by the ubiquitin-conjugating enzyme E2D3, ensuring the correct functioning of this protein import machinery.

## Introduction

Peroxisomes harbor a variety of enzymes involved in metabolic pathways such as beta-oxidation of fatty acids, plasmalogen synthesis, and detoxification of reactive oxygen species [1]. All these enzymes are synthesized on cytosolic ribosomes and posttranslationally targeted to the organelle [2]. The machinery that sorts these proteins to the peroxisomal matrix comprises more than a dozen components (reviewed in [3,4]). Mechanistically, these components can be grouped into 4 sets: (1) the shuttling receptor PEX5 plus its ancillary factor PEX7 [5–10]; (2) the docking/translocation module (DTM), a peroxisomal transmembrane complex through which newly synthesized proteins are translocated across the organelle membrane and that comprises PEX13, PEX14, and the RING-finger peroxins PEX2, PEX10, and PEX12 [11–13]; (3) the receptor export module (REM), which comprises PEX1 and PEX6 (2 mechanoenzymes of the “ATPases associated with diverse cellular activities” (AAA) family) and PEX26, a transmembrane protein that anchors the ATPases to the organelle membrane [14–17]; and (4) a set of soluble/cytosolic proteins involved in ubiquitination and deubiquitination of PEX5 [18–22].

The targeting pathway of newly synthesized proteins to the peroxisome matrix starts with their recognition in the cytosol by either PEX5 or a PEX5.PEX7 complex [5–10]. This activates the receptor for the next step of the pathway, i.e., the interaction of the PEX5-cargo protein complex with the peroxisomal DTM [23]. The DTM-PEX5 interaction is still ill defined, but it is known that after a reversible binding step (docking), PEX5 is inserted into the DTM acquiring a transmembrane topology [23–26]. Insertion of PEX5 into the DTM leads to the translocation of the cargo protein across the organelle membrane and culminates with the release of the cargo into the organelle matrix [24,27,28]. Interestingly, none of these steps requires energy from NTP hydrolysis—the driving force for the complete process resides in the high affinity/high avidity protein–protein interactions that are established between PEX5 on one side and components of the DTM on the other [24,27,29–32].

After releasing its cargo, PEX5 must be extracted from the DTM to initiate a new protein transport cycle. Extraction of PEX5 requires ATP hydrolysis and comprises 3 steps: first, PEX5 is monoubiquitinated at a phylogenetically conserved cysteine residue (cysteine 11 in the human protein) by the RING-finger peroxins of the DTM [18,19]; then, monoubiquitinated PEX5 (Ub-PEX5) is pulled from the DTM by the REM [15–17,33]; and, finally, Ub-PEX5 is deubiquitinated in the cytosol probably by a combination of enzymatic and non-enzymatic mechanisms [21,22,34].

An intriguing feature of the PEX5-mediated protein import pathway regards the type of ubiquitination with which PEX5 is modified at the DTM: A single ubiquitin is attached to a cysteine residue that is absolutely conserved in all PEX5 and PEX5-like proteins characterized up to now, from yeast to human [18,19,35,36]. The reason why a cysteine and not a lysine was maintained throughout evolution as the acceptor residue of the monoubiquitin remains enigmatic, particularly when we consider that engineered PEX5 proteins possessing a lysine at this position can still be monoubiquitinated at the DTM and extracted by the REM [34]. Two hypotheses were put forward to explain the nonconventional ubiquitination of PEX5 [34]. One proposes that the conserved cysteine residue in PEX5 might act as a redox switch to control protein import into the peroxisome. The other postulates that the susceptibility of the thioester bond in the Ub-PEX5 conjugate to cleavage by nucleophiles (e.g., glutathione) decreases the probability that cytosolic Ub-PEX5 is targeted to the proteasome for degradation. Some data supporting these 2 hypotheses were subsequently provided [37,38].

In addition to being monoubiquitinated at the conserved cysteine residue, PEX5 also undergoes other types of ubiquitination under specific experimental situations. For example, in yeast/fungi mutant strains lacking components of the REM (e.g., PEX1 or PEX6), PEX5 is modified with K48-linked oligoubiquitins at lysine residues near the conserved cysteine [39–41]. Through poorly comprehended mechanisms, these oligoubiquitinated receptors are then extracted from the DTM and targeted for proteasomal degradation.

Ubiquitination at a lysine residue has also been described for human PEX5 in cells subjected to oxidative stress. However, in this case the biological outcome is a much more drastic one. Indeed, it was proposed that ubiquitination of PEX5 at lysine 209 recruits the autophagy adaptor protein p62 to the peroxisome thus triggering the complete destruction of the organelle by selective autophagy, i.e., pexophagy [42]. The same phenomenon was proposed to occur in human cells harboring mutations in components of the REM [43], the most frequent cause of Zellweger syndrome [44], although neither the type of ubiquitination nor the PEX5 residue(s) targeted by this modification were defined in that study.

In this work, we used an established cell-free in vitro system [45] to further characterize ubiquitination of PEX5 at the DTM. We found that, in contrast to wild-type PEX5, a PEX5 protein in which the conserved cysteine residue was replaced by a lysine is robustly polyubiquitinated at the DTM when physiological concentrations of ubiquitin-activating enzyme (E1) and ubiquitin-conjugating enzyme (E2) are present in the assays. Importantly, polyubiquitinated PEX5 molecules are no longer optimal substrates for the REM, with PEX5 modified with long ubiquitin chains even becoming arrested at the DTM. Mechanistic analyses aiming at understanding why wild-type PEX5 is not polyubiquitinated revealed that ubiquitination at its cysteine 11 (Cys11) residue is a reversible and highly dynamic process. Remarkably, this reversibility does not involve a deubiquitinase or the chemical attack of the Ub-PEX5 thioester bond by a nucleophile such as glutathione. Rather, we found that the E2-catalyzed ubiquitination of PEX5 is a reversible reaction, with the E2-mediated deubiquitination step displaying faster kinetics than polyubiquitination of Ub-PEX5. This mechanism ensures that only monoubiquitinated PEX5 is produced at the DTM, and thus it avoids clogging of the DTM by polyubiquitinated PEX5 and, possibly, also the degradation of polyubiquitinated PEX5 by the proteasome.

## Results and discussion

### PEX5(C11K) is polyubiquitinated at the DTM when the REM is blocked

The peroxisomal protein import pathway can be fully recapitulated in vitro using a post-nuclear supernatant (PNS)-based system [45]. When radiolabeled PEX5 is used as a reporter protein in this system, steps such as the insertion of the cargo-loaded receptor into the peroxisomal DTM, its monoubiquitination at the conserved Cys11 residue and the subsequent ATP-dependent extraction of monoubiquitinated PEX5 (Ub-PEX5) back into the cytosol by the REM can all be easily monitored [17,19,23]. However, other types of PEX5 ubiquitination have not been detected in this system, thus far. Considering that the cytosolic protein concentration in a typical in vitro assay is approximately 44-fold lower than that found in vivo (4 g/L versus 175 g/L, respectively; see Materials and methods), we reasoned that this might reflect an insufficient amount of ubiquitin-activating (E1) and ubiquitin-conjugating (E2) enzymes in the assays. To test this, we performed in vitro assays using a PNS supplemented with physiological concentrations of recombinant E1 (0.5 μM; see Materials and methods) and E2D3 (2 μM; [46,47]), one of the E2s that mediates monoubiquitination of DTM-embedded PEX5 at its Cys11 [20]. For practical reasons, a ^35^S-labeled PEX5 protein possessing a lysine instead of a cysteine at position 11 (PEX5(C11K)) was used in these initial experiments. PEX5(C11K) is as functional as wild-type PEX5 in this in vitro system as well as in cellula, at least under overexpression conditions [34], but it presents the advantage of yielding a Ub-PEX5 species in which ubiquitin is linked to PEX5 through a stable isopeptide bond. Thus, Ub-PEX5(C11K), unlike the labile Ub-PEX5 thioester conjugate, can be analyzed using standard SDS-PAGE conditions (i.e., gels are run at room temperature and samples are treated with reducing agents before analysis, which increases resolution).

**Fig 1:**
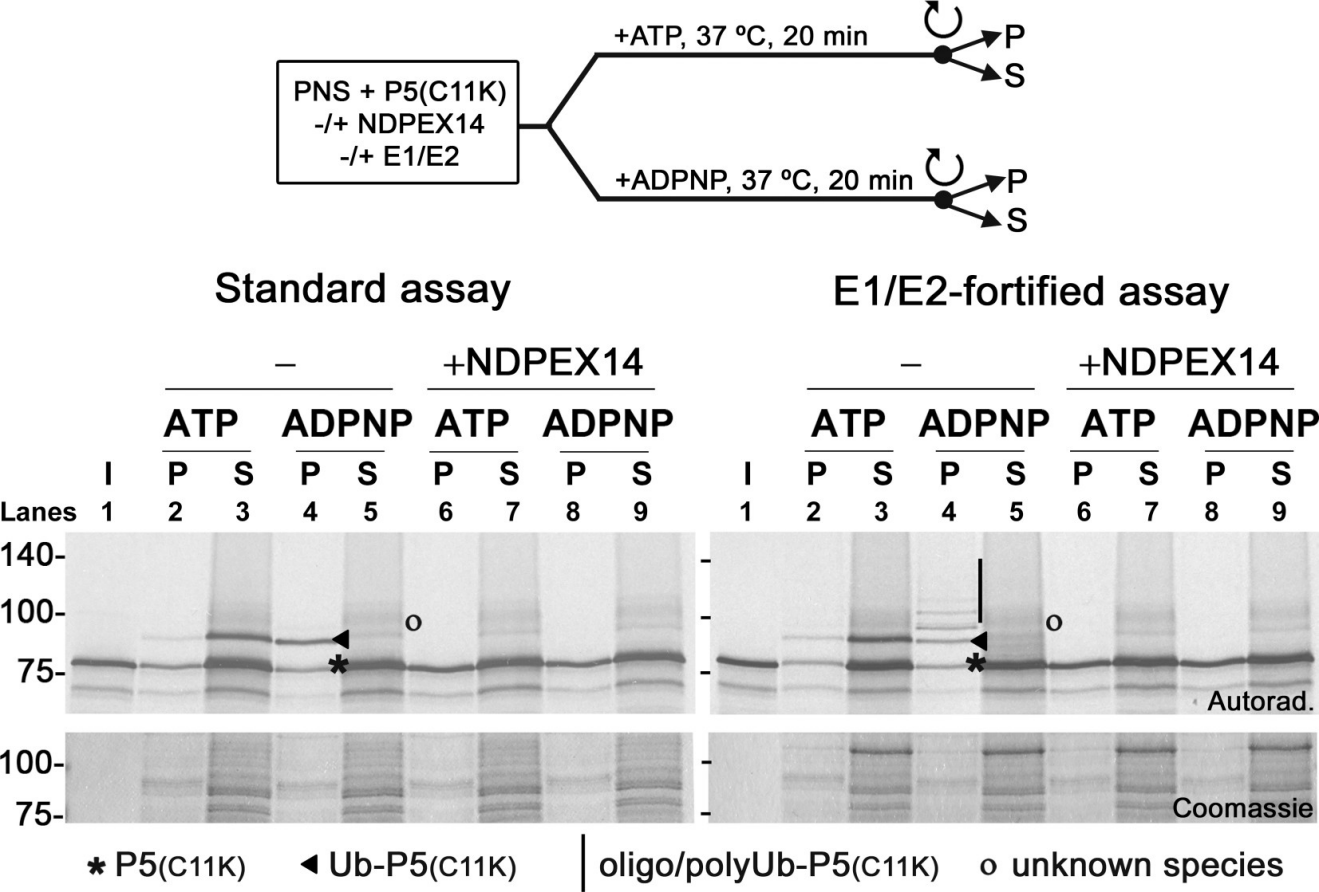
PEX5(C11K) is polyubiquitinated at the DTM when the REM is blocked. Radiolabeled PEX5(C11K) was subjected to standard (left panel) or E1/E2-fortified (right panel) single-step in vitro assays in the presence of either ATP or ADPNP, and in the absence or presence of NDPEX14 (lanes “-” and “+NDPEX14,” respectively). After treatment with NEM, reactions were centrifuged to isolate organelle (lanes “P”) and soluble (lanes “S”) proteins. Samples were analyzed by reducing SDS-PAGE/autoradiography. Note that recombinant NDPEX14, in addition to interacting strongly with soluble PEX5, also has some tendency to adsorb to membrane lipids [49]. This is probably the reason why more PEX5 is found in the organelle fractions in assays containing NDPEX14. Lanes I, ^35^S-PEX5(C11K) protein used in the assays; P5(C11K), Ub-P5(C11K) and oligo/polyUb-P5(C11K) indicate unmodified, mono-, and oligo/polyubiquitinated PEX5(C11K), respectively; “o”, PEX5(C11K) proteins of unknown identity (ubiquitinated?) that are occasionally detected in supernatants upon long exposures—their detection in the presence of ADPNP and/or recombinant NDPEX14 indicates that they are not engaged in the peroxisomal protein import pathway; numbers to the left indicate the molecular weight markers in kDa; autoradiographs (“Autorad.”) and Coomassie-stained dried gels (“Coomassie”) are shown. DTM, docking/translocation module; NEM, N-ethylmaleimide; REM, receptor export module.

Fig 1 shows the results of in vitro assays in which ^35^S-labeled PEX5(C11K) was incubated with a PNS supplemented or not with physiological amounts of E1 and E2, in the presence of either ATP or the non-hydrolysable ATP analog, ADPNP. A set of reactions containing NDPEX14, a soluble recombinant protein that comprises amino acid residues 1–80 of PEX14 (a DTM component; see Introduction), was also included in this experiment as a negative control. NDPEX14 binds with high affinity to the diaromatic motifs present in the N-terminal half PEX5 [32], which are required for insertion of the receptor into the DTM [31]. The ectopic presence of NDPEX14 in the soluble phase of the in vitro assays will, therefore, block insertion of PEX5 into the DTM, and thus ubiquitination of PEX5 by the RING E3 ligases of the DTM is no longer possible [45]. At the end of the incubation, reactions were centrifuged to separate organelles (lanes “P”) from soluble/cytosolic proteins (lanes “S”) and samples were analyzed by SDS-PAGE/autoradiography. In the absence of recombinant E1 and E2, a fraction of PEX5(C11K) was monoubiquitinated and appeared mainly in the supernatant of the ATP-supplemented assay (Fig 1, left panel, lane 3). As shown before [17,19,21], this means that PEX5(C11K) was (1) inserted into the peroxisomal DTM; (2) monoubiquitinated at its residue 11; and, finally (3) exported into the cytosol by the REM. In contrast, Ub-PEX5(C11K) remained in the organelle pellet of the assay supplemented with ADPNP (Fig 1, left panel, lane 4), as expected, because this ATP analog is efficiently used by the ubiquitin-activating E1 enzyme but is a potent inhibitor of the REM ([21,48]; see also Materials and methods). The assays performed with the E1/E2-fortified PNS revealed a similar result in the ATP condition (Fig 1, right panel, lanes 2 and 3). However, when the REM was blocked with ADPNP, several oligo/polyubiquitinated PEX5(C11K) species were now observed in the organelle fraction (Fig 1, right panel, lane 4). No ubiquitination of PEX5(C11K) was detected in assays containing NDPEX14 (Fig 1, left and right panels, lanes 6 to 9), indicating that only DTM-embedded PEX5 is a substrate for this modification. Apparently, the E3 ligase(s) that ubiquitinate(s) DTM-embedded PEX5 can modify the receptor with more than 1 ubiquitin molecule when extraction of peroxisomal Ub-PEX5 back into the cytosol is blocked.

### DTM-embedded PEX5(C11K) is polyubiquitinated at residue 11 yielding export-incompetent species

**Fig 2:**
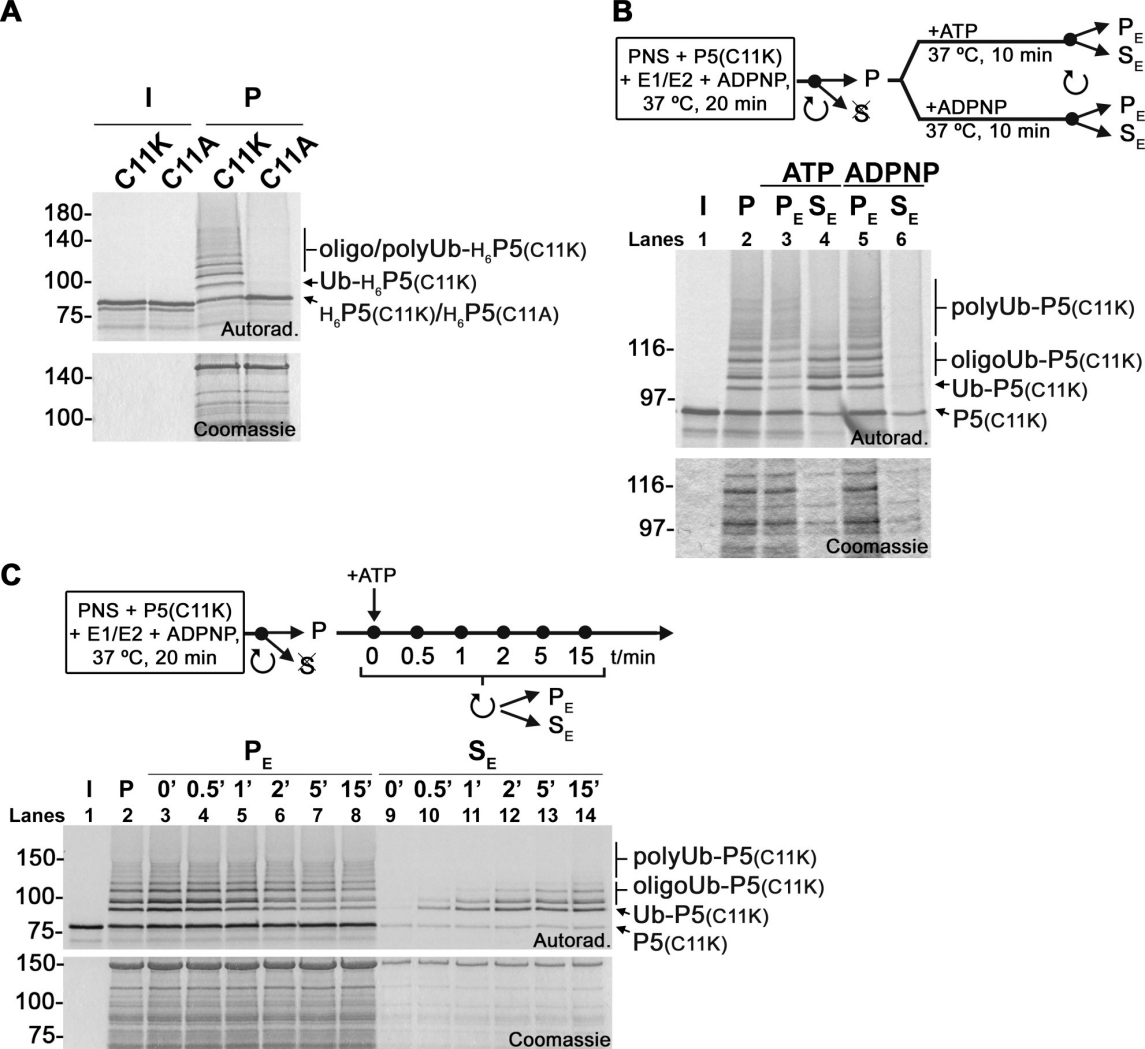
DTM-embedded PEX5(C11K) is polyubiquitinated at residue 11 yielding export-incompetent species. (A) ^35^S-His-PEX5(C11K) and ^35^S-His-PEX5(C11A) were subjected to E1/E2-fortified single-step in vitro assays in the presence of ADPNP. Organelles (lanes “P”) were isolated by centrifugation and analyzed by reducing SDS-PAGE/autoradiography. (B) PEX5(C11K) was subjected to an E1/E2-fortified two-step in vitro assay. After the first step, organelles (lane “P”) were isolated by centrifugation, resuspended in import buffer, and incubated (second step) in the presence of either ATP or ADPNP for 10 min. Organelle and soluble fractions (lanes “PE” and “SE,” respectively) were isolated and analyzed by reducing SDS-PAGE/autoradiography. (C) Export kinetics of ubiquitinated PEX5(C11K) species. PEX5(C11K)-containing organelles isolated from an E1/E2-fortified assay were resuspended in import buffer (lane “P”) and incubated at 37°C in the presence of ATP. Aliquots were withdrawn at the indicated time points and centrifuged to separate organelle (lanes “PE”) from soluble proteins (lanes “SE”). Samples were analyzed by reducing SDS-PAGE/autoradiography. Lanes I, radiolabeled PEX5 proteins used in the assays; H_6_P5(C11K) and H_6_P5(C11A) indicate unmodified His-tagged PEX5(C11K) and His-tagged PEX5(C11A), respectively; Ub- and oligo/polyUb-H_6_P5(C11K) indicates mono- and oligo/polyubiquitinated His-tagged PEX5(C11K); P5(C11K), Ub-P5(C11K), oligoUb-P5(C11K), and polyUb-P5(C11K) indicate unmodified, mono-, oligo-, and polyubiquitinated PEX5(C11K), respectively; numbers to the left indicate the molecular weight markers in kDa; autoradiographs (“Autorad.”) and Coomassie-stained dried gels (“Coomassie”) are shown. DTM, docking/translocation module.

Oligo/polyubiquitination of PEX5(C11K) could occur at its residue 11 or at other residues. To clarify this, we asked whether a PEX5 protein possessing a non-ubiquitinatable alanine at position 11 (PEX5(C11A)) is polyubiquitinated in the E1/E2-fortified in vitro assays. As shown in Fig 2A, no ubiquitinated forms of PEX5(C11A) were detected. This suggests that PEX5(C11K) is exclusively oligo/polyubiquitinated at its lysine 11.

We next asked whether oligo/polyubiquitinated PEX5(C11K) can still be extracted into the cytosol in an ATP-dependent manner as monoubiquitinated PEX5 is. A two-step in vitro assay [21] was used for this purpose. In the first step, we accumulated oligo/poly-Ub-PEX5(C11K) at the peroxisomal DTM using the E1/E2-fortified PNS assay, as described above. The organelles (“P”) were then isolated by centrifugation and resuspended. In the second step, the organelles were supplemented with either ATP or ADPNP, incubated at 37°C and again centrifuged to separate organelles (“PE”) from soluble proteins (“SE”). As shown in Fig 2B, several PEX5(C11K) ubiquitinated species were exported into the soluble phase of the reaction in an ATP-dependent manner (compare lanes 4 and 6). These include not only Ub-PEX5(C11K), as expected, but also PEX5(C11K) molecules containing 2 to 4–5 ubiquitins. Note that the exact size of the ubiquitin chains present in these export-competent PEX5(C11K) species is difficult to define due to the irregular spacing of the corresponding bands in the gels. For convenience, we will refer to these species simply as oligoubiquitinated PEX5(C11K). Importantly, PEX5(C11K) molecules modified with a larger number of ubiquitins (hereafter referred to as polyubiquitinated PEX5(C11K)) remained in the organelle fraction (Fig 2B, lane 3). The differences in the export efficiencies of the different PEX5 species are better perceived in a time course assay. As shown in Fig 2C, monoubiquitinated PEX5 is rapidly exported into the soluble phase of the assay in an ATP-dependent manner, as described before [28,50]. Oligoubiquitinated PEX5 species are also exported although with a clear delayed kinetics, whereas polyubiquitinated PEX5 remained in the organelle fraction during the complete time course. Thus, the longer the ubiquitin chain attached to a PEX5 molecule the worse is its export competence.

### Wild-type DTM-embedded PEX5 is not polyubiquitinated when the REM is blocked

**Fig 3:**
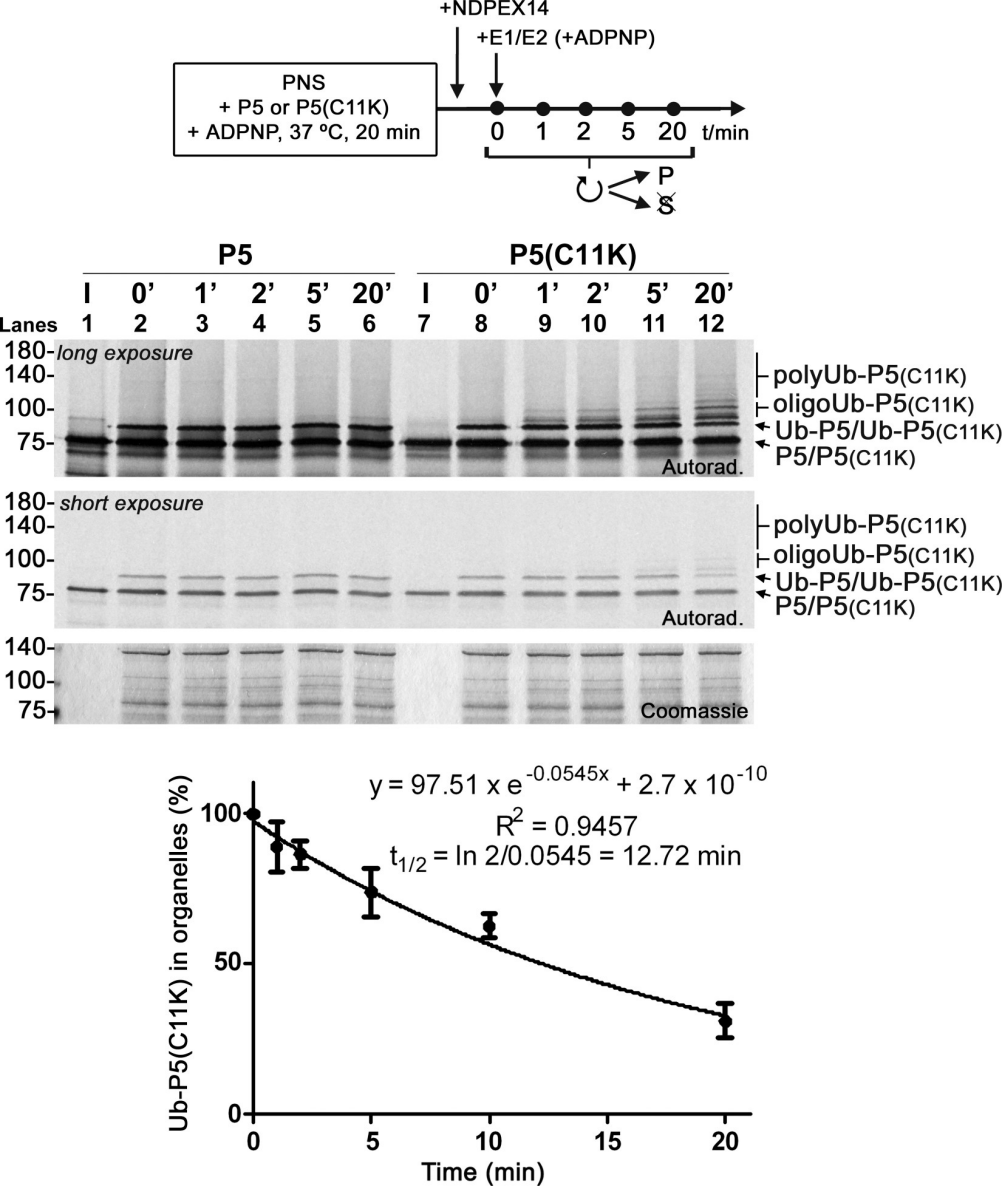
Wild-type DTM-embedded PEX5 is not polyubiquitinated when the REM is blocked. Radiolabeled PEX5 and PEX5(C11K) were incubated with a PNS under standard conditions in the presence of ADPNP for 20 min. After adding recombinant NDPEX14 and incubating for 2 min (to stop further insertion of PEX5 proteins into the DTM), pre-charged E1/E2 were then added to the PNSs and incubation proceeded at 37°C. Aliquots were removed at the indicated time points, treated with NEM, and organelles were isolated by centrifugation. Samples were analyzed by nonreducing SDS-PAGE/autoradiography. Lanes I, ^35^S-PEX5 proteins used in the assays; P5 and Ub-P5, and P5(C11K), Ub-P5(C11K), oligoUb-P5(C11K), and polyUb-P5(C11K) indicate unmodified and monoubiquitinated PEX5, and unmodified, mono-, oligo-, and polyubiquitinated PEX5(C11K), respectively; numbers to the left indicate the molecular weight markers in kDa; long- and short-exposure autoradiographs (“Autorad.”) and the Coomassie-stained dried gel (“Coomassie”) are shown. Lower panel, the graph shows the amount of monoubiquitinated PEX5(C11K) (percentage of Ub-PEX5(C11K)) in organelles over time; a one phase exponential decay model was fitted to the data (averages and standard deviations of 4 experiments performed on different days with 2 independent PNSs are presented; t_1/2_, half-life). The data underlying the graph shown in the figure can be found in S1 Data. DTM, docking/translocation module; NEM, N-ethylmaleimide; PNS, post-nuclear supernatant; REM, receptor export module.

We then asked whether the findings described above for PEX5(C11K) can be extended also to wild-type PEX5. Fig 3 shows a time-course experiment in which PNSs containing peroxisomal monoubiquitinated PEX5 or PEX5(C11K) (obtained in a standard assay in the presence of ADPNP) were supplemented with recombinant E1/E2 and further incubated in the presence of ADPNP to maintain the REM blocked. This experiment revealed that trace amounts of PEX5(C11K) species containing 2 to 4 ubiquitin molecules can be detected just 1 min after adding the E1/E2 enzymes (Fig 3, lane 9) and that more than half of Ub-PEX5(C11K) is further ubiquitinated by the end of the 20-min incubation (Fig 3, lane 12). Assuming that conversion of Ub-PEX5(C11K) to oligo/polyubiquitinated PEX5 species can be treated as a pseudo-first order event (i.e., that oligo/polyubiquitination depends linearly on the concentration of Ub-PEX5(C11K) and that the concentration of ubiquitin-charged E2D3 enzyme remains constant), we estimated a half-life of approximately 13 min for Ub-PEX5(C11K) in these assays (Fig 3, lower panel). To our surprise, although the amounts of monoubiquitinated PEX5 and monoubiquitinated PEX5(C11K) were similar at the 0 min time point (Fig 3, lanes 2 and 8, respectively), almost no oligo/polyubiquitinated PEX5 could be detected at any time point analyzed. Seemingly, oligo/polyubiquitin chains cannot be efficiently attached to, or retained by, a monoubiquitinated wild-type PEX5 protein. The experiments below address the mechanism behind this PEX5 property.

### The side-chain length of residue 11 of PEX5 is not important for its polyubiquitination

**Fig 4:**
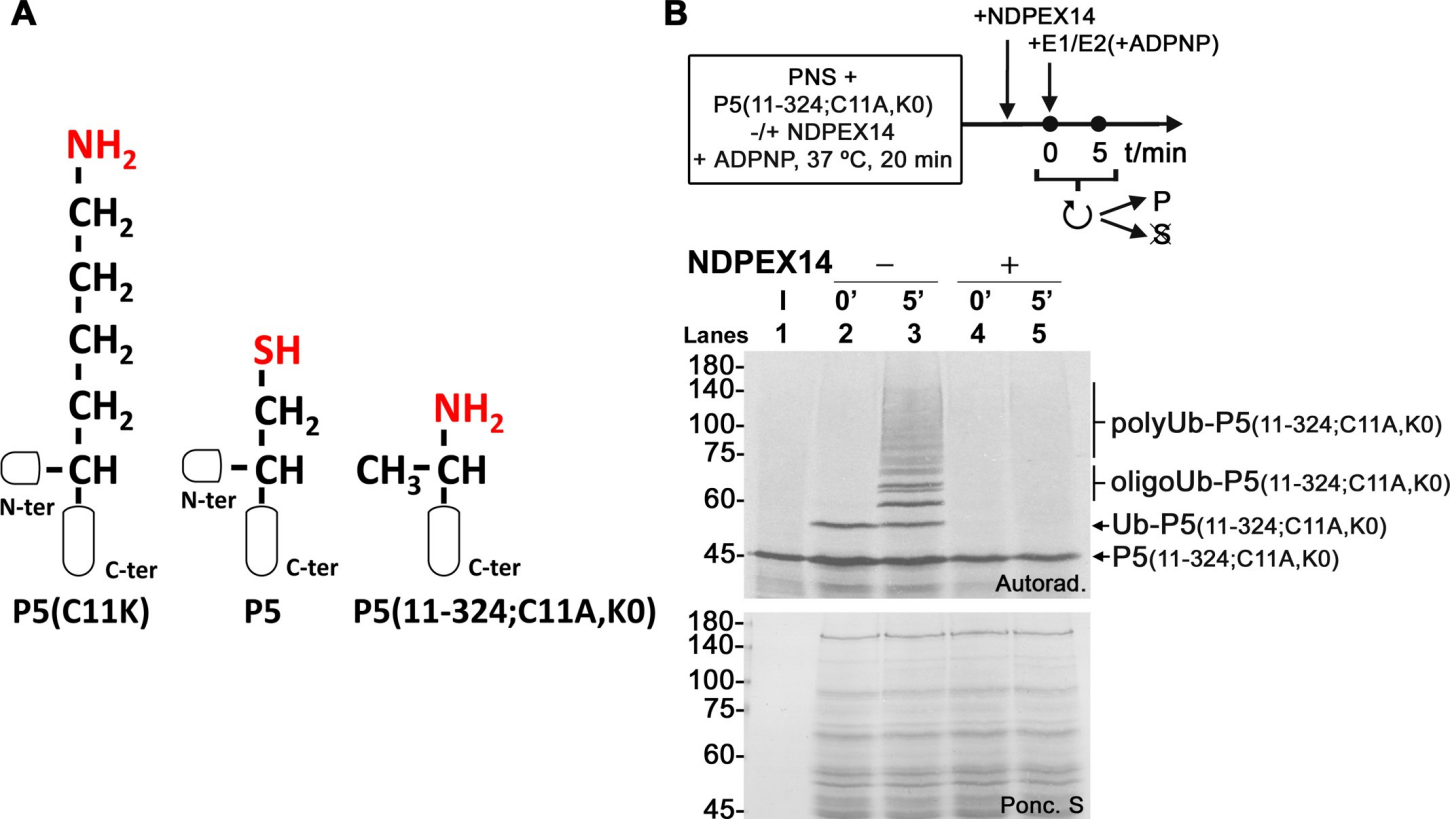
The side-chain length of residue 11 of PEX5 is not important for its polyubiquitination. (A) Schematic representation of the ubiquitin acceptor chemical group (shown in red) in PEX5(C11K) (P5(C11K)), wild-type PEX5 (P5), and PEX5(11–324;C11A,K0) (P5(11–324;C11A,K0)) proteins. N-ter and C-ter, N- and C-termini, respectively. (B) Radiolabeled PEX5(11–324;C11A,K0) was incubated with a PNS in the presence of ADPNP for 20 min at 37°C, either in the absence or presence of NDPEX14 (lanes “-” and “+NDPEX14,” respectively). After this incubation, an additional amount of NDPEX14 was added to both reactions, and aliquots were removed for analysis (lanes “0’”). Pre-charged recombinant E1/E2 were then added to the assays and incubation at 37°C proceeded for 5 min (lanes “5’”). Organelles were isolated by centrifugation and analyzed by SDS-PAGE/autoradiography. Lane I, ^35^S-PEX5 protein used in the assays; P5(11–324;C11A,K0), Ub-P5(11–324;C11A,K0), oligoUb-P5(11–324;C11A,K0), and polyUb-P5(11–324;C11A,K0), indicate unmodified, mono-, oligo-, and polyubiquitinated PEX5(11–324;C11A,K0), respectively; numbers to the left indicate the molecular weight markers in kDa; autoradiographs (“Autorad.”) and the Ponceau S-stained membrane are shown (“Ponc. S”). PNS, post-nuclear supernatant.

In principle, a ubiquitin molecule linked to lysine 11 of DTM-embedded PEX5(C11K) protrudes into the cytosol a few more angstroms than a ubiquitin molecule linked to cysteine 11 of wild-type PEX5 (see Fig 4A). Such a structural difference might be crucial for the E3 ubiquitin ligase that adds additional ubiquitins to DTM-embedded Ub-PEX5 thus explaining why Ub-PEX5(C11K) is oligo/polyubiquitinated, whereas Ub-PEX5 is not. Data suggesting that this is not the case were obtained in assays performed with an engineered PEX5 protein, PEX5(11–324;C11A,K0), that comprises amino acid residues 11–324 of PEX5, contains no lysines and possesses a free alpha-amino group at residue 11 of PEX5, which is an alanine instead of a cysteine in this protein. Despite all these modifications, we have shown recently that PEX5(11–324;C11A,K0) is monoubiquitinated at its alpha-amino group yielding an extraction-competent species [33]. As shown in Fig 4B, this protein is also polyubiquitinated in the E1/E2-fortified in vitro system, suggesting that the type of ubiquitination detected in these experiments—mono- or oligo/polyubiquitination—is not determined by the structural properties of residue 11 of PEX5.

### Ubiquitination of PEX5 at the conserved cysteine residue is a reversible reaction that avoids overubiquitination of PEX5

Replacement of cysteine 11 by a lysine in PEX5 also changes the type of covalent bond that links PEX5 to ubiquitin. In the Ub-PEX5(C11K) conjugate, this is a stable isopeptide bond, whereas in Ub-PEX5 the 2 moieties are linked by a labile thioester bond. Thus, we next asked whether the inability of PEX5 to hold oligo/polyubiquitin chains at its Cys11 residue might be due to the lability of the ubiquitin-PEX5 bond. Specifically, we hypothesized that no oligo/polyubiquitin chains would be retained by PEX5 simply because the rate at which PEX5 loses a ubiquitin chain and is monoubiquitinated de novo would be higher than the rate at which monoubiquitinated PEX5 is oligo/polyubiquitinated.

Several different mechanisms could lead to the fast deubiquitination of peroxisomal PEX5. An obvious one regards the fact that ubiquitin thioester conjugates are promptly disrupted by thiol-containing molecules such as glutathione (GSH). In fact, we have shown previously that the half-life of peroxisomal Ub-PEX5 in isolated organelles (i.e., in the absence of cytosolic components) is approximately 10 min in the presence of 5 mM GSH [34]. The concentration of GSH in the assays described above was just 2 mM and thus the half-life of the thioester bond in the Ub-PEX5 conjugate should be approximately 25 min, a value that is too high to explain why oligo/polyubiquitinated PEX5 was not detected. Nevertheless, we still considered the possibility that oligo/polyubiquitinated PEX5 species might be particularly susceptible to GSH or to some other component present in the cytosolic fraction, which, as stated above, was not present in the previously reported experiments [34]. However, as shown in S1 Fig (see also S1 File), assays performed in the absence of GSH and cytosolic components yielded the same results, i.e., peroxisomal PEX5(C11K) was oligo/polyubiquitinated, whereas PEX5 was not.

In contrast to conventional ubiquitination in which a thioester-linked ubiquitin is transferred from an E2 to a lysine residue of a substrate through an essentially irreversible reaction, thiol-thioester exchange reactions are typically reversible [51]. We reasoned that if this were the case for ubiquitination of wild-type PEX5, then it would explain why DTM-embedded PEX5 is unable to retain a ubiquitin chain: any ubiquitin chain adventitiously built on Cys11 of PEX5 would be rapidly transferred to an uncharged E2 molecule and thus diluted manifold in a large pool of soluble E2D3 molecules. To test this possibility, we started by asking whether monoubiquitination of wild-type PEX5 at the DTM is indeed a reversible reaction.

**Fig 5:**
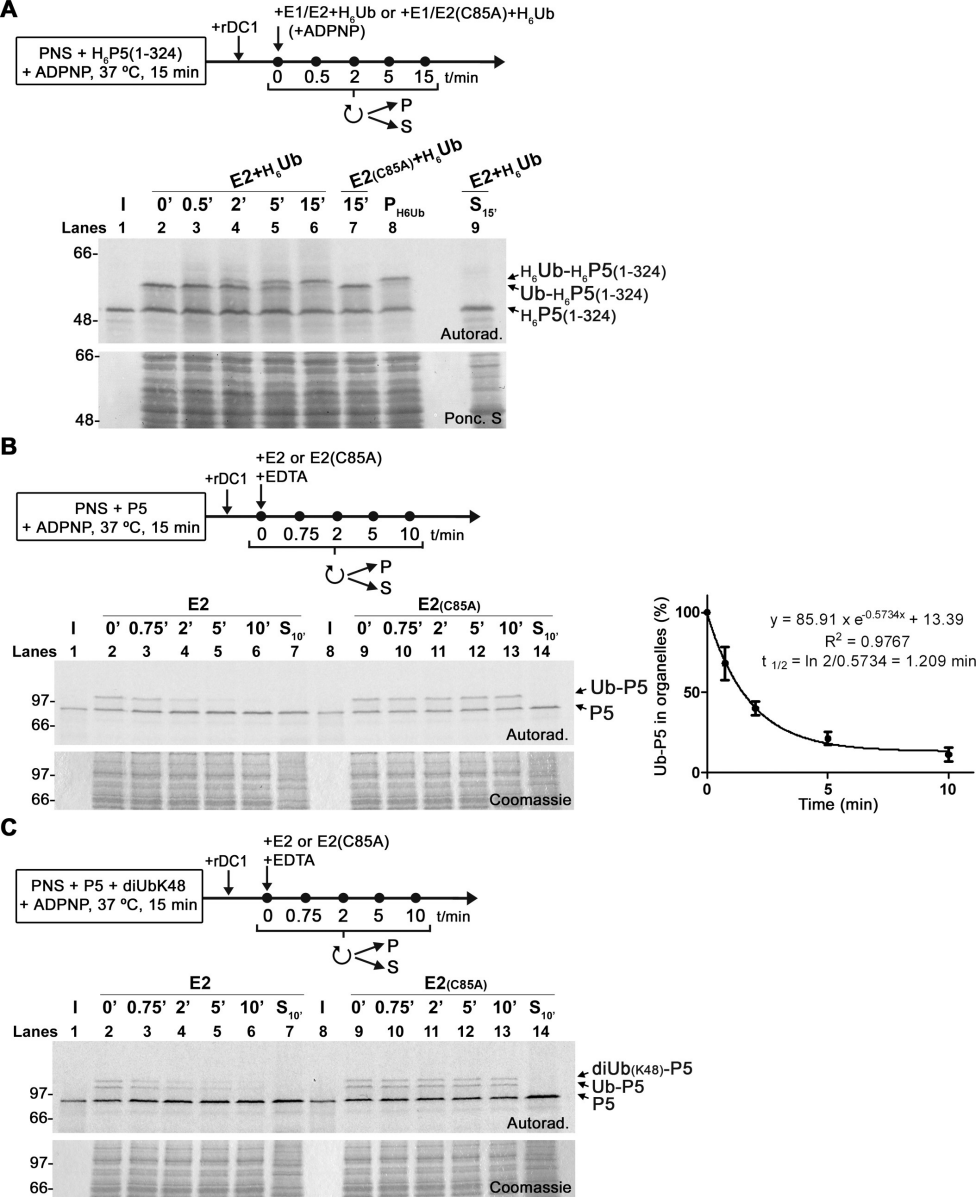
Ubiquitination of PEX5 at the conserved cysteine residue is a reversible reaction. (A) ^35^S-labeled His-PEX5(1–324) was subjected to an in vitro ubiquitin exchange assay comprising 2 steps. In the first step, the radiolabeled protein was incubated with a PNS for 15 min at 37°C in the presence of ADPNP and 2 μM of bovine ubiquitin. After inhibiting further insertion of the PEX5 protein with recombinant PEX5(1–324) (rDC1), an aliquot was withdrawn (lane 2, “0’”). In the second step, one fraction of the reaction was diluted 10-fold in buffer containing recombinant E1/E2 pre-charged with His-Ub and aliquots were withdrawn at the indicated time points (lanes 3–6, 9). Another fraction received instead E1/E2(C85A) pre-incubated with His-Ub (lane 7). An organelle pellet containing radiolabeled His-PEX5(1–324) monoubiquitinated with His-Ub instead of Ub was also loaded (lane 8, “P_H6Ub_”) to show the electrophoretic migration of the corresponding conjugate. Organelle (“P”) and soluble (“S”) fractions (lanes 2–8 and lane 9, respectively) were analyzed. (B) Left panel, E2-mediated deubiquitination of DTM-embedded Ub-PEX5. Radiolabeled PEX5 was incubated with a PNS in the presence of ADPNP. After stopping further insertion/ubiquitination of PEX5 with recombinant PEX5(1–324) (rDC1) and EDTA, the PNS was diluted 10-fold in buffer containing EDTA plus 2 μM of either E2 or E2(C85A). Aliquots were withdrawn at the indicated time points and centrifuged to obtain organelle (“P”; lanes 2–6 and 9–13) and soluble (S_10’_) fractions. Right panel, the graph shows the amount of monoubiquitinated PEX5 (percentage of Ub-PEX5) in organelles over time; a one phase exponential decay model was fitted to the data (averages and standard deviations of 3 experiments performed on different days with the same PNS are presented; t_1/2_, half-life). The data underlying the graph shown in the figure can be found in S1 Data. (C) E2-mediated deubiquitination of PEX5 modified with a di-ubiquitin(K48) chain. As in (B), with the exception that 5 μM of di-Ub(K48) instead of Ub was used in the first step of the assay. Lanes I, ^35^S-PEX5 protein used in the assays; H_6_P5(1–324), Ub-H_6_P5(1–324), and H_6_Ub-H_6_P5(1–324), indicate unmodified his-tagged PEX5(1–324) and his-tagged PEX5(1–324) modified with ubiquitin and His-Ub, respectively; P5, Ub-P5, and diUb_(K48)_-P5, indicate unmodified PEX5 and PEX5 modified with Ub or di-Ub(K48), respectively; samples were analyzed by nonreducing SDS-PAGE/autoradiography; numbers to the left indicate the molecular weight markers in kDa; autoradiographs (“Autorad.”), the Coomassie-stained dried gels (“Coomassie”), and the Ponceau S-stained membrane (“Ponc. S”) are shown. PNS, post-nuclear supernatant.

In one approach, a PNS containing DTM-embedded monoubiquitinated PEX5 (obtained using standard conditions, i.e., in the presence of ADPNP and endogenous ubiquitin-activating and ubiquitin-conjugating enzymes) was diluted 10-fold in buffer containing ADPNP and recombinant E1/E2 previously charged with hexa-histidine-tagged ubiquitin. Aliquots were withdrawn at different time points and organelle pellets were analyzed by SDS-PAGE/autoradiography. As shown in Fig 5A, approximately half of the ubiquitin bound to PEX5 disappeared after 5 min of incubation being substituted by the His-tagged ubiquitin species.

In another approach, a PNS containing DTM-embedded Ub-PEX5 (obtained as above) was simply diluted 10-fold in buffer containing 2 μM uncharged recombinant E2D3 and 20 mM EDTA to stop de novo ubiquitination by endogenous E1. As shown in Fig 5B, Ub-PEX5 was rapidly deubiquitinated displaying a half-life of about 1.2 min (Fig 5B, right panel). Importantly, a control assay using a catalytically inactive version of E2D3, E2D3(C85A), showed that deubiquitination of PEX5 is a specific phenomenon that requires an active E2 (Fig 5B, lanes 8 to 14). An identical experiment performed with PEX5(C11K) revealed that this protein is not deubiquitinated when incubated with uncharged E2D3, as expected (see S2A Fig and S1 File).

Different results were obtained when soluble/cytosolic Ub-PEX5 obtained from an ATP-supplemented reaction (see Materials and methods) was subjected to the same treatments. In this case, no deubiquitination of Ub-PEX5 was observed after incubation with recombinant E2D3 or E2D3(C85A) (see S2B Fig and S1 File). Thus, only DTM-embedded Ub-PEX5 is deubiquitinated by E2D3, strongly suggesting that the reaction also involves the E3 ubiquitin ligase(s) of the DTM.

Finally, to determine whether wild-type PEX5 modified with a ubiquitin chain can also lose its chain in an E2D3-mediated process, we explored the fact that DTM-embedded PEX5 can be modified with oligoubiquitin chains at its residue 11 when large amounts of these chains are added to the in vitro assays [33]. Fig 5C shows an assay in which PEX5 was modified with a K48-linked diubiquitin chain and, subsequently, incubated with either recombinant E2D3 or E2D3(C85A), as described above. The data show that incubation with E2D3, but not E2D3(C85A), resulted in the fast deubiquitination of peroxisomal PEX5.

**Fig 6:**
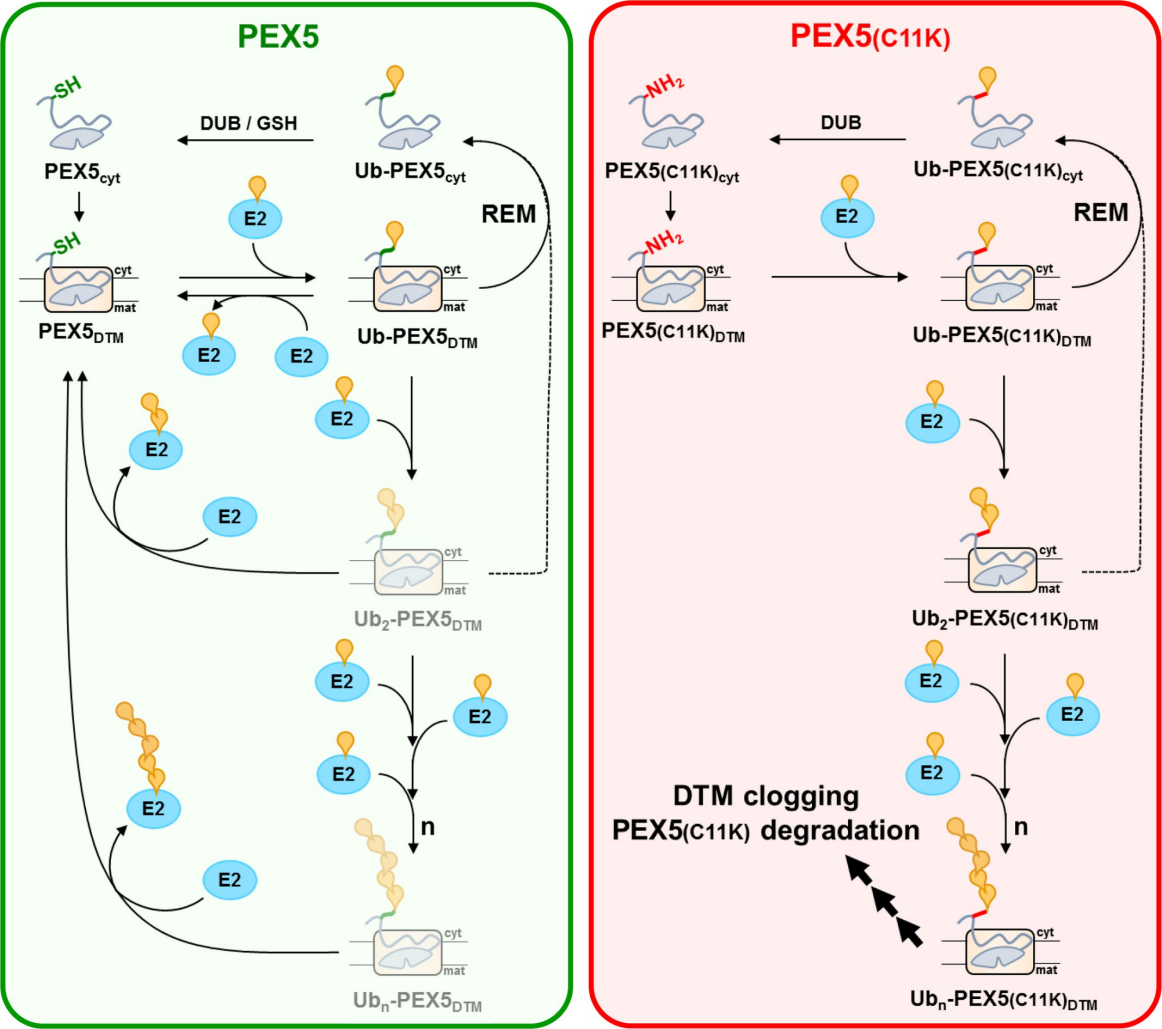
Reversibility of PEX5 ubiquitination avoids overubiquitination of PEX5 at the DTM. Left panel—Cytosolic cargo-loaded PEX5 (PEX5_cyt_) gets inserted into the DTM thus delivering its cargo into the organelle matrix. After cargo translocation, DTM-embedded PEX5 is monoubiquitinated at a conserved cysteine residue (Ub-PEX5_DTM_) and is then extracted from the DTM by the REM. In the cytosol, Ub-PEX5_cyt_ is deubiquitinated either enzymatically by a deubiquitinase (DUB) or via a thiol-thioester exchange with glutathione (GSH) [34], and a new protein transport cycle begins. When Ub-PEX5_DTM_ is not promptly extracted, it may slowly undergo additional ubiquitination yielding oligo/polyubiquitinated species (Ub_n_-PEX5_DTM_). However, the oligo/polyubiquitin chains built on cysteine 11 of PEX5 are rapidly transferred to uncharged E2, thus generating non-ubiquitinated PEX5_DTM_. According to this model, the steady-state amounts of these oligo/polyubiquitinated PEX5_DTM_ species (light gray forms) are extremely low. Right panel—PEX5(C11K) behaves similarly to wild-type PEX5 with the exception that ubiquitination at its lysine 11 residue is an irreversible reaction. Thus, any oligo/polyubiquitin adventitiously built on lysine 11 cannot be transferred to an uncharged E2. Oligo/polyubiquitinated PEX5(C11K) species are poor REM substrates leading to DTM clogging. Also, oligo/polyubiquitinated PEX5(C11K) may undergo proteasomal degradation. Cyt, cytosol; DUB, deubiquitinase; DTM, docking/translocation module; E2, ubiquitin-conjugating enzyme; GSH, glutathione; mat, matrix; PEX5/PEX5(C11K)_cyt_, cytosolic PEX5 or PEX5(C11K), respectively; PEX5/PEX5(C11K)_DTM_, DTM-embedded PEX5 or PEX5(C11K), respectively; REM, receptor export module; Ub, ubiquitin; Ub-/Ub_2_-/Ub_n_-PEX5/PEX5(C11K)_DTM_, DTM-embedded mono-/oligo-/polyubiquitinated PEX5 or PEX5(C11K), respectively; Ub-PEX5/PEX5(C11K)_cyt_, cytosolic monoubiquitinated PEX5 or PEX5(C11K), respectively.

Altogether, the data above indicate that ubiquitination of PEX5 at the conserved cysteine residue occurs through a reversible reaction (see Fig 6). Reversibility of PEX5 ubiquitination places the DTM-embedded receptor in a highly dynamic equilibrium with the uncharged and ubiquitin-charged pools of cytosolic E2D3. This equilibrium in turn ensures that any polyubiquitin chain adventitiously built at Cys11 of PEX5 is rapidly removed by an uncharged E2, and thus diluted into the large cytosolic E2 pool. It is relevant to note that members of the E2D family are found mostly in the uncharged state in vivo [47,52].

The advantage of having a mechanism that ensures that PEX5 is not overubiquitinated at the DTM is probably 2-fold. First, as shown in Fig 2B and 2C, polyubiquitinated PEX5 species are no longer substrates for the REM, and thus a mechanism that avoids polyubiquitination of PEX5 also avoids DTM jamming. Second, considering that oligo/polyubiquitins can recruit or be recruited to the proteasome, a mechanism that saves PEX5 from overubiquitination should also save PEX5 from premature degradation. Although we did not address this possibility here, some data suggesting that this may be true for yeast PEX5 are already available [37]. Indeed, the steady-state levels of PEX5(C6K) (the yeast equivalent of human PEX5(C11K)) in a yeast strain lacking the deubiquitinase UBP15 are lower than those of wild-type PEX5 but can be normalized by growing cells in the presence of MG132, a proteasome inhibitor [37].

In addition to the advantages referred to above, it might also be possible that by avoiding polyubiquitination of PEX5 at the DTM, the mechanism described here also saves peroxisomes from premature pexophagy (see Introduction). However, we note that previous experiments with PEX5 knock-out mouse embryonic fibroblasts complemented with either PEX5 or PEX5(C11K) did not reveal noticeable differences in peroxisome numbers [34]. Also, quantification of pexophagy in *Saccharomyces cerevisiae* cells expressing endogenous levels of either PEX5 or PEX5(C6K) using a Pex11-GFP breakdown assay [53] did not reveal differences between the 2 strains (see S3 Fig and S1 File). Thus, if polyubiquitination of PEX5(C6K) at the DTM has some effect on pexophagy, this effect is, at best, a minor one.

In this work, we show that the nonconventional ubiquitination of PEX5 at the DTM is a reversible reaction depending on a catalysis-competent E2. The reversibility kinetics of this reaction explains why wild-type PEX5 cannot retain an oligo/polyubiquitin at its cysteine residue. By avoiding overubiquitination of PEX5, this mechanism maintains the DTM available for multiple rounds of protein import and, possibly, also saves PEX5 from proteasomal degradation.

An important question that remains unanswered regards the identity of the E3 ligase that catalyzes the E2-mediated ubiquitination/deubiquitination of PEX5 at the DTM. According to recent structural data, the 3 RING peroxins of the DTM are organized into 2 functional units: (1) the monomeric RING domain of PEX2; and (2) the heterodimer comprising the RING domains of PEX10 and PEX12 [13]. It has been hypothesized that PEX2 catalyzes monoubiquitination of PEX5 at the conserved cysteine residue, and thus, it is likely that the E2-mediated deubiquitination of Ub-PEX5 is also promoted by PEX2. However, our data are also compatible with a mechanism in which both PEX2 and the PEX10.PEX12 dimer play a role in PEX5 ubiquitination/deubiquitination, with one promoting monoubiquitination of PEX5 and the other catalyzing its deubiquitination. Further work is necessary to clarify this issue.

Finally, it is worth noting that ubiquitination at protein cysteine residues is not a peculiarity of peroxisomal protein import receptors. Indeed, a number of different proteins that are also subjected to this type of unconventional ubiquitination are presently known [54,55]. Although most cases reported in the literature link ubiquitination at cysteine residues to proteasomal degradation, suggesting that those proteins are polyubiquitinated (e.g., [56]), there is at least 1 example in which ubiquitination at cysteine residues has a regulatory (non-degradative) role. This is the histone methyltransferase Suv39h1 which is monoubiquitinated at cysteine residues, a modification that probably leads to the chromatin eviction of the methyltransferase from the IκBα locus, thus inducing expression of this NFκB inhibitor [57]. Whether the mechanism described here for PEX5 monoubiquitination can be transposed to Suv39h1 (and other proteins) is presently unknown, but this is surely a possibility to consider in any future studies on ubiquitination at cysteine residues.

## Materials and methods

### Ethics statement

Male Wistar Han rats were handled according to the EU Directive 2010/63/EU and the national Decree-law number 113–2013, and the protocols were approved by the Portuguese Veterinarian Board (DGAV) and the i3S Ethical Committee (authorization JA_2019_01 –“Biogénese e função de proteínas/enzimas envolvidas em modificações pós-traducionais, na produção de ROS e na importação de proteínas peroxissomais”).

### Plasmids

The following plasmids were described before: (1) pET28a-His-PEX5—encodes an N-terminally hexa-histidine (His)-tagged version of the large isoform of human PEX5 [30]; (2) pET28a-His-PEX5(C11A)—encodes a version of the large isoform of human PEX5 possessing an alanine at position 11 [30]; (3) pET28a-His-PEX5(C11K)—encodes a variant of the latter protein possessing a lysine at position 11 [33]; (4) pET23a-PEX5(C11K)—encodes an untagged version of the latter protein [33]; (5) pET28a-His-PEX5(1–324)—encodes a C-terminally truncated protein comprising residues 1–324 of the large isoform of PEX5 preceded by an His-tag [20]; (6) pET28a-His-Ub-PEX5(11–324;C11A,K0)—encodes a PEX5 fusion protein comprising His-tagged ubiquitin followed by amino acid residues 11–324 of a PEX5 variant possessing an alanine at position 11 and with all its lysine residues replaced by arginine residues [33]; (7) pQE30-His-PEX14(1–80)—encodes an N-terminally His-tagged truncated PEX14 protein comprising residues 1–80 of human PEX14, referred to as NDPEX14 [58]; (8) pET28b-His-mE1—encodes an N-terminally His-tagged version of mouse E1-activating enzyme [59]; (9) pET28a-His-E2D3—encodes an N-terminally His-tagged version of human E2D3 (also known as UbcH5c; [33]); (10) pOPINs-USP21—encodes a protein comprising a His-SUMO tag followed by the catalytic domain of human USP21 (Addgene plasmid # 61585; [60]; a gift from David Komander), obtained from the Addgene repository.

The following plasmids were produced:

1. pET23a-PEX5—encodes an untagged version of the large isoform of human PEX5. It was obtained by cloning the NdeI/SalI fragment derived from pET28a-His-PEX5 [30] into the NdeI/SalI restriction sites of pET23a (Novagen);
2. pET28a-His-E2D3(C85A)—encodes an N-terminally His-tagged version of an active site mutant of the human E2D3; it possesses an alanine at position 85 instead of the catalytic cysteine [61]. It was obtained by site-directed mutagenesis (Quik-Change II site-directed mutagenesis kit, Agilent Technologies) using the plasmid pET28a-His-E2D3 [33] and the primers 5′-GTAATGGCAGCATTGCGCTCGATATTCTAAGATCAC-3′ and 5′-GTGATCTTAGAATATCGAGCGCAATGCTGCCATTAC-3′;
3. pET28a-His-Ub—encodes an N-terminally His-tagged human ubiquitin. The cDNA was synthesized and cloned into the NdeI/EcoRI restriction sites of pET28a (Novagen) by GenScript.

### Expression and purification of recombinant proteins

Recombinant NDPEX14 [58], His-PEX5(1–324) [58], and His-mE1 [59] were obtained as previously described with the following modifications: NDPEX14 was stored in 50 mM Tris-HCl (pH 8.0), 150 mM NaCl, 1 mM EDTA-NaOH (pH 8.0); His-PEX5(1–324) was stored in 50 mM Tris-HCl (pH 8.0), and His-mE1 was stored in 10 mM Tris-HCl (pH 8.0), 1 mM EDTA-NaOH (pH 8.0), 1 mM tris(2-carboxyethyl)phosphine.

His-PEX5(315–639) ([58]; also referred to as TPRs), His-SUMO-cUSP21, His-E2D3, and His-E2D3(C85A) were obtained exactly as described before [33]. The di-ubiquitin chain linked through K48 was obtained through linear solid phase peptide synthesis, as previously described [62].

The N-terminally His-tagged version of human ubiquitin was expressed in *E*. *coli* BL21(DE3) for 3 h at 37°C with 1 mM IPTG (isopropyl β-D-1-thiogalactopyranoside), and purified using Ni Sepharose 6 Fast Flow affinity chromatography resin (GE Healthcare) according to the manufacturer instructions. His-Ub was stored in 20 mM Tris-HCl (pH 7.5), 150 mM NaCl, 0.5 mM tris(2-carboxyethyl)phosphine.

Aliquots of all recombinant proteins were stored at −80°C.

### In vitro synthesis of radiolabeled proteins

Radiolabeled proteins were synthesized in vitro using the TNT T7 Quick Coupled Transcription/Translation System (Promega) in the presence of EasyTag L-[^35^S]methionine (specific activity > 1,000 Ci/mmol, Perkin Elmer) for 90 min at 30°C, according to the manufacturer instructions. Aliquots of the rabbit reticulocyte lysates containing the radiolabeled proteins were snap-frozen in liquid nitrogen and stored at −80°C. Radiolabeled His-Ub-PEX5(11–324;C11A,K0) was cleaved with His-SUMO-cUSP21 before in vitro assays, as described before [33].

### Cell-free in vitro assays

Male Wistar Han rats were maintained under standard conditions, and rats with 6 to 10 weeks of age were fasted overnight, euthanized, and their livers extracted for preparation of rat liver PNSs for in vitro cell-free assays, as described previously [45].

Both single- and two-step in vitro assays were performed in this work [21,45]. A standard in vitro single-step assay (total volume of 100 μl) consists in the incubation of 600 μg of PNS protein (previously primed by incubating at 37°C for 4 min with 0.3 mM ATP) with 0.5 to 1 μl of ^35^S-PEX5 (or the indicated PEX5 variant) for 15 to 20 min at 37°C in import buffer (50 mM MOPS-KOH (pH 7.4), 0.25 M sucrose, 50 mM KCl, 5 mM MgCl_2_, 20 μM methionine, 2 μg/ml of N-(trans-epoxysuccinyl)-L-leucine-4-guanidinobutylamide (E-64)) supplemented with 2 mM GSH, 2 to 5 μM ubiquitin aldehyde (Ubal; [21,63]), 2 to 20 μM bovine ubiquitin (Ub), and either 3 mM ATP or ADPNP, a non-hydrolysable ATP analog. Note that the imido group that connects the β and γ phosphates in ADPNP renders this compound essentially hydrolysis-resistant for most ATPases but does not affect much ATP-dependent ligases that cleave ATP between the α and β phosphates. Thus, ADPNP strongly inhibits the REM (an ATPase; [21]) while being a reasonably good substrate for the E1 ubiquitin-activating enzyme (a ligase; [48]). Where specified, in vitro assays were supplemented with a solution containing recombinant His-mE1 and His-E2D3 (0.5 μM and 2 μM final concentrations, respectively) that had been previously incubated 4 min at 37°C in the presence of 20 μM ubiquitin or His-Ub, 3 μM Ubal, 1 mM ADPNP and, unless otherwise noted, 2 mM GSH. In the ubiquitin exchange assays, the final concentration of E1 in the assays was decreased to 0.05 μM. Where specified, association/insertion of ^35^S-PEX5 into the peroxisomal DTM was blocked by adding either 10 μM NDPEX14 [45,64] or 5 μM of recombinant His-PEX5(1–324) (to compete with the radiolabeled protein for DTM binding/insertion; [24]). In the E2-mediated deubiquitination assays, after accumulating Ub-PEX5 or di-Ub(K48)-PEX5 at the organelles and blocking further insertion of PEX5 with recombinant His-PEX5(1–324), reactions were diluted 1:10 (v/v) with pre-warmed (37°C) import buffer supplemented with 1 μM Ubal, 20 mM EDTA-NaOH (pH 8.0), and either 2 μM His-E2D3 or His-E2D3(C85A). At the end of the 37°C incubations, reactions were placed on ice, treated with 25 mM NEM (final concentration) for 5 min, and diluted 1:10 (v/v) with ice-cold SEMK buffer (0.25 M sucrose, 20 mM MOPS-KOH (pH 7.4), 1 mM EDTA-NaOH (pH 8.0), 80 mM KCl). Organelle (“P”) and soluble fractions (“S”) were isolated by centrifugation at 16,000 *x g* for 20 min at 4°C, and processed for SDS-PAGE/autoradiography, under reducing or nonreducing conditions (see below).

Two-step in vitro assays [21,33] were used to monitor the ATP-dependent extraction of mono/oligo/polyubiquitinated PEX5 proteins and to obtain soluble Ub-PEX5 for the E2-mediated deubiquitination assays. In the first step, the radiolabeled PEX5 protein was incubated for 20 min at 37°C with a primed PNS in import buffer containing 3 mM ADPNP, 2 mM GSH, 5 μM Ubal, 10 to 20 μM Ub, and, where indicated, pre-charged E1 and E2. After incubation, the organelles were isolated by centrifugation and the soluble fraction discarded, as described above, and carefully resuspended in ice-cold import buffer containing 2 μM Ubal, 1 μM of His-PEX5(1–324) (in some experiments, 0.15 μM bovine serum albumin (BSA) was also included), and either 3 mM ATP or ADPNP, and subjected to a second incubation at 37°C. Organelle suspensions were then placed on ice, treated with 25 mM NEM (final concentration), and diluted 1:10 (v/v) with ice-cold SEMK buffer. After centrifugation as above, to separate organelles (“PE”) from soluble proteins (“SE”), and precipitation of soluble proteins with 10% TCA, samples were processed for SDS-PAGE. In the export kinetics assays, organelles from the first step were carefully resuspended in import buffer containing 0.1 mM ADPNP, and the organelle suspension was placed at 37°C for 2 min. Export of ubiquitinated PEX5 species was initiated by adding a pre-warmed solution containing (values in parenthesis are the final concentrations in the assays): ATP (5 mM), Ubal (2 μM), BSA (0.15 μM), and His-PEX5(1–324) (1 μM). Aliquots were withdrawn at the indicated time points, centrifuged and processed for SDS-PAGE/autoradiography. To generate soluble Ub-PEX5 for the E2-mediated deubiquitination assays, the two-step in vitro assays were centrifuged immediately after the second incubation (i.e., the NEM treatment and the SEMK dilution steps were omitted) and the supernatant containing Ub-PEX5 was collected and subjected to E2-mediated deubiquitination assays, as described above. All experiments were performed at least 3 times, unless indicated otherwise.

### Miscellaneous

The concentration of rat liver cytosolic proteins in the in vitro assays was estimated considering that cytosolic proteins represent approximately 2/3 of the PNS protein. The concentration of Uba1 in liver cytosol was estimated considering that 60 micrograms of mouse liver cytosol contain approximately 20 ng of E1 [59] and that protein concentration in the cytosol is 175 g/L [65].

Samples for reducing and nonreducing SDS-PAGE were incubated 30 min at 37°C with agitation, in Laemmli sample buffer (50 mM Tris-HCl (pH 6.8), 2% (w/v) SDS, 0.167% (w/v) bromophenol blue, 10% (v/v) glycerol, and 2 mM EDTA-NaOH (pH 8.0)) containing 100 mM DTT or 20 mM NEM, respectively. Reducing and nonreducing SDS-gels were run at room temperature and 4°C, respectively.

### Image processing and densitometric analysis

All autoradiographs, Coomassie-stained dried gels, and Ponceau S-stained membranes were scanned using an HP Scanjet 4850 scanner. Figures were assembled using Adobe Photoshop (version 11.0). When necessary, linear contrast adjustments were made.

To obtain the half-life of monoubiquitinated PEX5 in reversibility and polyubiquitination experiments, the amounts of monoubiquitinated PEX5 in the organelles at each time point were quantified using the Fiji/ImageJ software version 1.53t [66]. The amount of monoubiquitinated PEX5 at time point “0” was set as 100%. One phase exponential decay equations were fitted to the data using the GraphPad Prism 6 (version 6.01) with the constraint plateau >0.

### Experiments with *Saccharomyces cerevisiae*

#### (1) Yeast growth

Yeast cells were grown at 30°C in either of the following mediums: minimal medium 2 (YM2) (2% (w/v) glucose, 0.17% (w/v) yeast nitrogen base without amino acids and ammonium sulfate, 0.5% (w/v) ammonium sulfate, 1% (w/v) casamino acids) or minimal medium 1 (YM1) for the selection of all prototrophic markers (2% (w/v) glucose, 0.17% (w/v) yeast nitrogen base without amino acids and ammonium sulfate, 0.5% (w/v) ammonium sulfate). As carbon source, 2% (w/v) glucose was added unless otherwise stated. Overnight cultures did not reach the stationary phase before they were diluted to an OD_600_ of 0.1 in the morning in YM2 2% (w/v) glucose medium and grown until OD_600_ = 1 (Log phase cultures). For post-log phase cells, an overnight culture was incubated till OD_600_ = 6–8 was reached without further addition of fresh medium. For induction of peroxisome proliferation, overnight cultures were diluted in YM1 containing 0.3% (w/v) glucose, (uracil and leucine-deficient) and grown during the day for 7 to 8 h before cells were transferred to YM2 lacking glucose but containing 0.12% (v/v) oleate, 0.2% (v/v) Tween 40, 0.1% (w/v) yeast extract and 1:10 (v/v) diluted and grown overnight for 16 h. Pexophagy was induced by collecting the cells through centrifugation (1,000 × *g*, 5 min) and resuspending the cell pellet to 1 ml of starvation medium per 10 OD_600_ units. Starvation medium lacks a nitrogen source (SD-N; 0.17% (w/v) yeast nitrogen base without amino acids and ammonium sulfate, 2% (w/v) glucose) [67,68]. The appropriate amino acid stocks were added to minimal medium as required. In all, 10 OD_600_ units were collected at the selected time points as indicated in the figure. Cells were either analyzed by western blotting or by fluorescence microscopy.

#### (2) Yeast strain construction

*S*. *cerevisiae* strains used in this study are BY4742 (*MATα his3Δ1 leu2Δ0 lys2Δ0 ura3Δ0*) obtained from the EUROSCARF consortium or the *pex1*::kanMX4 and *pex5*::kanMX4 derivatives thereof.

The plasmids used in this study are derivatives of the *URA3* and *LEU2* centromeric plasmids Ycplac33 and Ycplac111 [69]. These ARS1/CEN4 plasmids are present at 1 to 2 copies per cell [70]. Pex11-GFP (pEH005) has been described previously [71]. The *PEX5* ORF flanked by 607 bp upstream and 339 downstream sequence was amplified from genomic DNA using primers VIP4766 and VIP4767 (S1 Table) and cloned into Ycplac111 digested with Kpn1 and Pst1 through the gap repair in yeast [72]. The *pex5C6K* mutant was subsequently generated through site-directed mutagenesis using oligonucleotides VIP4768 and VIP4769 (S1 Table). The Pex5 sequences were confirmed by Sanger sequencing of both strands. For pexophagy assays, *pex5*::kanMX4 cells were transformed with pEH005 (Pex11-GFP) and either Ycplac111-PEX5 (WT) or Ycplac111-*pex5C6K*. *pex1*::kanMX4 were transformed with pEH005 and Ycplac111 (empty plasmid).

#### (3) Western blot analyses of yeast proteins

For preparation of extracts by alkaline lysis, 10 OD_600_ units of cells were harvested, and cell pellets resuspended in 0.2M NaOH and 0.2% (v/v) β-mercaptoethanol and left on ice for 10 min. Soluble protein was precipitated by adding 5% (w/v) TCA and incubating on ice for at least 15 min. Following centrifugation (13,000 × *g*, 5 min, 4°C), the pellet was resuspended in 10 μl 1 M Tris-HCl (pH 9.4), and 90 μl 1× SDS-PAGE sample loading buffer was added and samples were boiled for 10 min at 95°C. Samples (1 OD_600_ equivalent) were resolved by SDS-PAGE followed by immunoblotting. Blots were blocked in 2% (w/v) fat-free Marvel milk in TBS-Tween-20 (50 mM Tris-HCl (pH 7.6), 150 mM NaCl, 0.1% (v/v) Tween-20). GFP-tagged proteins were detected using monoclonal anti-GFP (mouse IgG monoclonal antibody clone 7.1 and 13.1; 1:3,000 (v/v) dilution; Roche, cat. no. 11814460001). Pgk1 was detected by a monoclonal anti-Pgk1 antibody (mouse; 1:7,000 (v/v) dilution; Invitrogen, cat. no. 459250). Secondary antibody was HRP-linked anti-mouse polyclonal (goat; 1:4,000 (v/v) dilution; Bio-Rad). Detection was achieved using enhanced chemiluminescence reagents (GE Healthcare) and chemiluminescence imaging.

#### (4) Image acquisition

Yeast cells were analyzed with an Axiovert 200M microscope (Carl Zeiss) equipped with an Exfo X-cite 120 excitation light source, band pass filters (Carl Zeiss and Chroma Technology), a Plan-Apochromat 63×1.4 NA objective lens (Carl Zeiss) and a digital camera (Orca ER; Hamamatsu Photonics). Image acquisition was performed using Volocity software (PerkinElmer). Fluorescence images were collected as 0.5 μm z-stacks, merged into 1 plane as maximum intensity projections using Openlab software (PerkinElmer) and processed further in Photoshop (Adobe). Bright-field images were collected in 1 focal plane and processed to highlight the circumference of the cells in blue. Each imaging experiment was performed at least 3 times, and representative images are shown. For quantitation, 1 experiment was used.

## Supporting information

S1 Table Primers used to generate the *pex5C6K* yeast strain. Sequences of both primers are presented in the 5’ → 3’ orientation.
(DOCX)

S1 Fig DTM-embedded wild-type PEX5 is not oligo/polyubiquitinated in the absence of GSH and cytosolic proteins. Radiolabeled PEX5 and PEX5(C11K) were subjected to two-step polyubiquitination assays. In the first step, a primed PNS was incubated with ^35^S-PEX5 (lanes “P5”) or ^35^S-PEX5(C11K) (lanes “P5(C11K)”) in import buffer containing ADPNP and Ubal (but no GSH) for 20 min. After stopping further import with NDPEX14, the organelles (“P”) were isolated by centrifugation, and resuspended in import buffer lacking GSH (lanes “0’”). Organelle suspensions were subjected to a second incubation in the presence of ADPNP and recombinant E1 and E2 pre-charged with Ub in the absence of GSH. Aliquots were removed at the indicated time points and treated with NEM. The organelles (“PE”) were isolated by centrifugation and analyzed by nonreducing SDS-PAGE/autoradiography. Lanes I, ^35^S-PEX5 proteins used in the assays; P5/P5(C11K) and Ub-P5/Ub-P5(C11K) indicate unmodified and monoubiquitinated PEX5 or PEX5(C11K), respectively; oligoUb-P5(C11K) and polyUb-P5(C11K) indicate oligo- and polyubiquitinated PEX5(C11K), respectively; numbers to the left indicate the molecular weight markers in kDa; long- and short-exposure autoradiographs (“Autorad.”) and the Coomassie-stained dried gel (“Coomassie”) are shown.
(TIF)

S2 Fig DTM-embedded monoubiquitinated-PEX5(C11K) and cytosolic/soluble monoubiquitinated wild-type PEX5 are not deubiquitinated by the E2. (A) Radiolabeled PEX5(C11K) was incubated with a PNS in the presence of ADPNP. After stopping further insertion/ubiquitination of PEX5 with recombinant PEX5(1–324) (rDC1) and EDTA, the PNS was diluted 10-fold in buffer containing EDTA plus 2 μM of either E2 or E2(C85A). Aliquots were withdrawn at the indicated time points and centrifuged to obtain organelle (“P”; lanes 2–6 and 9–13) and soluble (“S_10’_”) fractions (lanes 7 and 14) (*n* = 2). (B) Radiolabeled PEX5 was incubated with a PNS in the presence of ADPNP to accumulate monoubiquitinated PEX5 at peroxisomes. Organelles were isolated, resuspended (lanes 2 and 8, “P”), and subjected to a second incubation in the presence of ATP to extract Ub-PEX5 from the DTM. Organelles (PE) and soluble (SE) fractions were then isolated by centrifugation, and soluble Ub-PEX5 was incubated with either E2 (lanes 4–6) or E2 (C85A) (lanes 10–12), as described in Fig 5B (main text). Aliquots were withdrawn at the indicated time points. Lanes I, ^35^S-PEX5 proteins used in the assays; P5(C11K) and Ub-P5(C11K), indicate unmodified and monoubiquitinated PEX5(C11K), respectively; P5 and Ub-P5, indicate unmodified and monoubiquitinated PEX5, respectively; samples were analyzed by nonreducing SDS-PAGE/autoradiography; numbers to the left indicate the molecular weight markers in kDa; the autoradiographs (“Autorad.”) and Coomassie-stained dried gels (“Coomassie”) are shown.
(TIF)

S3 Fig PEX5C6K does not affect pexophagy. (A–D) Cultures of the strains indicated expressing Pex11-GFP were grown for 16 h on oleate medium (ole) before being transferred to nitrogen starvation conditions (starvation) for the time indicated (2–22 h). (A) Pexophagy was monitored by western blot analysis to detect Pex11-GFP and its breakdown products. GFP* indicates the relative protease-resistant degradation products that temporarily accumulate and are indicative of vacuolar breakdown. Detection of Pgk1 was used to verify equal loading. (B) Pex11-GFP full length and breakdown products observed after 6 h starvation were quantified using ImageJ and expressed as percentage of the total band intensity (*n* = 4). Two-tailed Student’s *t* test revealed that there was no statistically significant (ns) difference between Pex5 WT and *pex5C6K* cells. (C) Epifluorescence microscopy analysis of the strains indicated after growth on oleate (Ole 16 h) and 6 h nitrogen starvation (starvation (6 h)). GFP signal is projected as a merge Z stack, bright-field images were collected in one plane and shown as separate panels on the right and also processed to highlight the cell circumference in blue and overlaid the Z-stack GFP projection. (D) Quantitation of peroxisome number from a representative experiment shown in (C) using Kruskal–Wallis analysis test. Scale bar, 5 μm. (E) Western blot analysis of logarithmically and post-logarithmically growing cultures of the strains indicated expressing Pex11-GFP and analyzed with western blotting using monoclonal anti-GFP and anti-Pgk1. The data underlying the graphs shown in the figure can be found in S1 Data.
(TIF)

S1 File Supporting information text. (DOCX)

S1 Raw Images Uncropped original gels/blots of Figs 1, 2A, 2B, 2C, 3, 4B, 5A, 5B, 5C, S1, S2A, S2B, S3A and S3E. The molecular weights of the ladder in kiloDaltons (kDa) are shown for each blot.
(PDF)

S1 Data PDFs of the excel files containing all the raw data for Figs 3, 5B, S3B and S3D. (PDF)

## Abbreviations

AAA: ATPases associated with diverse cellular activities
ADPNP, 5′-adenylyl-beta: gamma-imidodiphosphate (also known as adenosine 5′-(β,γ-imido)triphosphate
BSA: bovine serum albumin
DTM: docking/translocation module
E1: ubiquitin-activating enzyme
E2: ubiquitin-conjugating enzyme
GSH: glutathione
NDPEX14: N-terminal domain of PEX14
NEM: N-ethylmaleimide
PNS: post-nuclear supernatant
REM: receptor export module
Ub-PEX5: monoubiquitinated PEX5
Ubal: ubiquitin aldehyde
