# Supplementary material for: Noncanonical and reversible cysteine ubiquitination prevents the overubiquitination of PEX5 at the peroxisomal membrane
Source: PLoS Biol. 2024 Mar 12;22(3):e3002567. doi: 10.1371/journal.pbio.3002567 (PMC10959387; doi:10.1371/journal.pbio.3002567)
Supplement: S1 Table — Sequences of both primers are presented in the 5’ → 3’ orientation. (DOCX) [file pbio.3002567.s001.docx]

**S1 Table. Primers used to generate the *pex5C6K* yeast strain.** Sequences of both primers are presented in the 5’ → 3’ orientation.

| Primer code | Sequence 5'-3' |
| --- | --- |
| VIP4766 | GCCAGTGAATTCGAGCTCGGTACCATAGGATCATATCCGCTCAGAG |
| VIP4767 | GCCAAGCTTGCATGCCTGCAGCCCTGATAAACCTCCTTTGGC |
| VIP4768 | CACATGGACGTAGGAAGTaaaTCAGTGGGAAATAATCC |
| VIP4769 | GGATTATTTCCCACTGAtttACTTCCTACGTCCATGTG |
