# Supplementary figures and images for: Noncanonical and reversible cysteine ubiquitination prevents the overubiquitination of PEX5 at the peroxisomal membrane
Source: PLoS Biol. 2024 Mar 12;22(3):e3002567. doi: 10.1371/journal.pbio.3002567 (PMC10959387; doi:10.1371/journal.pbio.3002567)

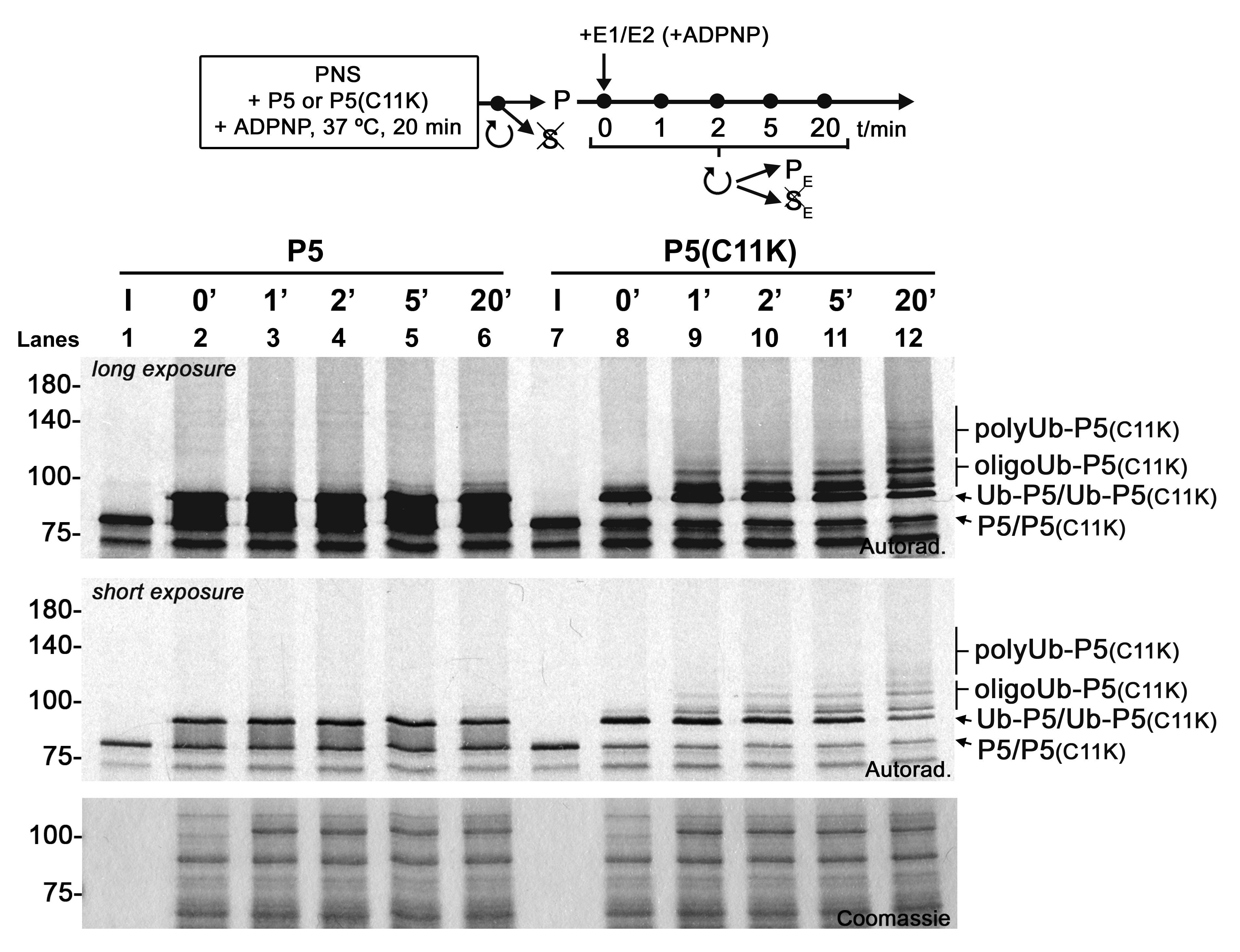

Supplement: S1 Fig — Radiolabeled PEX5 and PEX5(C11K) were subjected to two-step polyubiquitination assays. In the first step, a primed PNS was incubated with 35S-PEX5 (lanes “P5”) or 35S-PEX5(C11K) (lanes “P5(C11K)”) in import buffer containing ADPNP and Ubal (but no GSH) for 20 min. After stopping further import with NDPEX14, the organelles (“P”) were isolated by centrifugation, and resuspended in import buffer lacking GSH (lanes “0’”). Organelle suspensions were subjected to a second incubation in the presence of ADPNP and recombinant E1 and E2 pre-charged with Ub in the absence of GSH. Aliquots were removed at the indicated time points and treated with NEM. The organelles (“PE”) were isolated by centrifugation and analyzed by nonreducing SDS-PAGE/autoradiography. Lanes I, 35S-PEX5 proteins used in the assays; P5/P5(C11K) and Ub-P5/Ub-P5(C11K) indicate unmodified and monoubiquitinated PEX5 or PEX5(C11K), respectively; oligoUb-P5(C11K) and polyUb-P5(C11K) indicate oligo- and polyubiquitinated PEX5(C11K), respectively; numbers to the left indicate the molecular weight markers in kDa; long- and short-exposure autoradiographs (“Autorad.”) and the Coomassie-stained dried gel (“Coomassie”) are shown. (TIF) [file pbio.3002567.s002.tif]

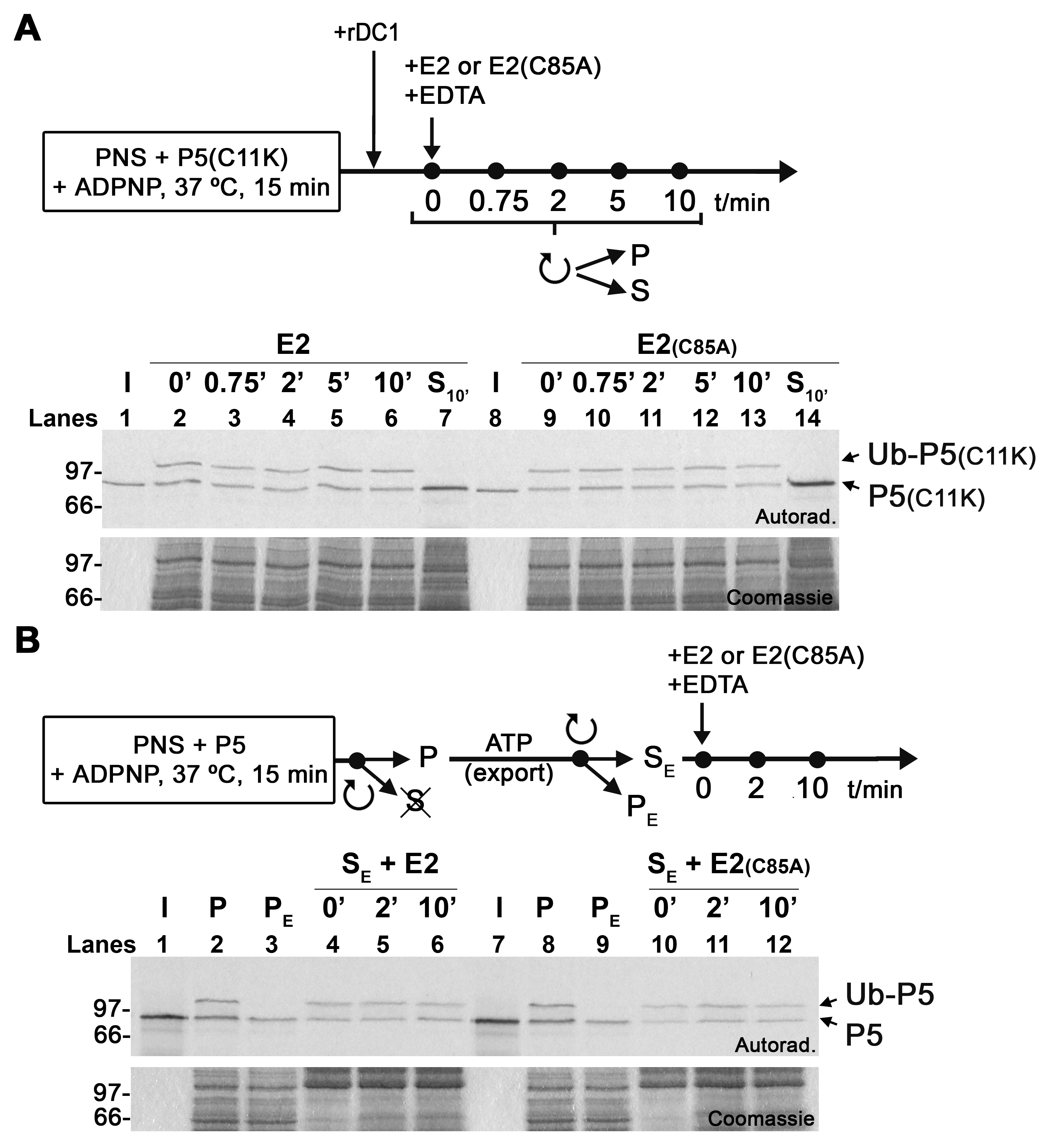

Supplement: S2 Fig — (A) Radiolabeled PEX5(C11K) was incubated with a PNS in the presence of ADPNP. After stopping further insertion/ubiquitination of PEX5 with recombinant PEX5(1–324) (rDC1) and EDTA, the PNS was diluted 10-fold in buffer containing EDTA plus 2 μM of either E2 or E2(C85A). Aliquots were withdrawn at the indicated time points and centrifuged to obtain organelle (“P”; lanes 2–6 and 9–13) and soluble (“S10’”) fractions (lanes 7 and 14) (n = 2). (B) Radiolabeled PEX5 was incubated with a PNS in the presence of ADPNP to accumulate monoubiquitinated PEX5 at peroxisomes. Organelles were isolated, resuspended (lanes 2 and 8, “P”), and subjected to a second incubation in the presence of ATP to extract Ub-PEX5 from the DTM. Organelles (PE) and soluble (SE) fractions were then isolated by centrifugation, and soluble Ub-PEX5 was incubated with either E2 (lanes 4–6) or E2 (C85A) (lanes 10–12), as described in Fig 5B (main text). Aliquots were withdrawn at the indicated time points. Lanes I, 35S-PEX5 proteins used in the assays; P5(C11K) and Ub-P5(C11K), indicate unmodified and monoubiquitinated PEX5(C11K), respectively; P5 and Ub-P5, indicate unmodified and monoubiquitinated PEX5, respectively; samples were analyzed by nonreducing SDS-PAGE/autoradiography; numbers to the left indicate the molecular weight markers in kDa; the autoradiographs (“Autorad.”) and Coomassie-stained dried gels (“Coomassie”) are shown. (TIF) [file pbio.3002567.s003.tif]

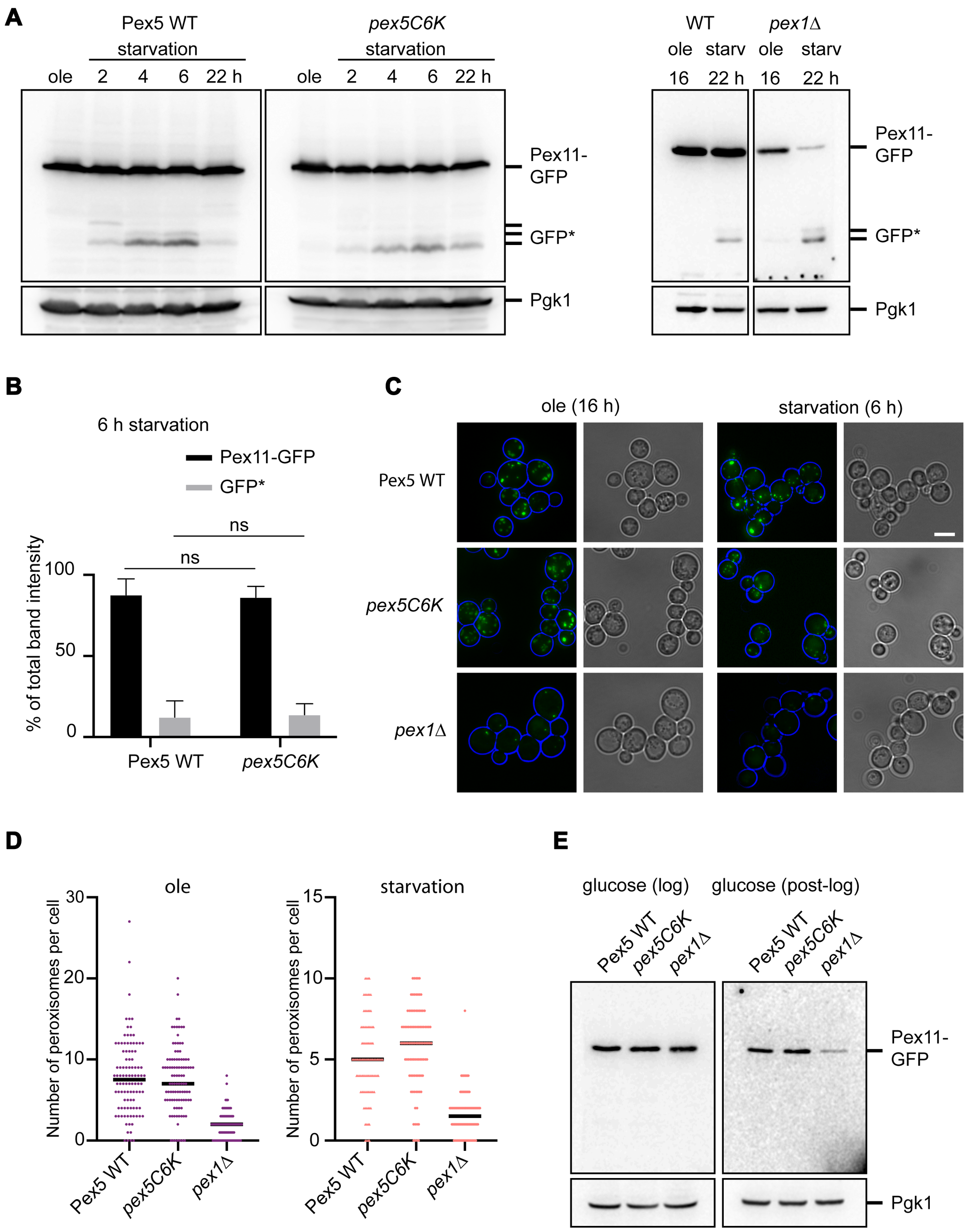

Supplement: S3 Fig — (A–D) Cultures of the strains indicated expressing Pex11-GFP were grown for 16 h on oleate medium (ole) before being transferred to nitrogen starvation conditions (starvation) for the time indicated (2–22 h). (A) Pexophagy was monitored by western blot analysis to detect Pex11-GFP and its breakdown products. GFP* indicates the relative protease-resistant degradation products that temporarily accumulate and are indicative of vacuolar breakdown. Detection of Pgk1 was used to verify equal loading. (B) Pex11-GFP full length and breakdown products observed after 6 h starvation were quantified using ImageJ and expressed as percentage of the total band intensity (n = 4). Two-tailed Student’s t test revealed that there was no statistically significant (ns) difference between Pex5 WT and pex5C6K cells. (C) Epifluorescence microscopy analysis of the strains indicated after growth on oleate (Ole 16 h) and 6 h nitrogen starvation (starvation (6 h)). GFP signal is projected as a merge Z stack, bright-field images were collected in one plane and shown as separate panels on the right and also processed to highlight the cell circumference in blue and overlaid the Z-stack GFP projection. (D) Quantitation of peroxisome number from a representative experiment shown in (C) using Kruskal–Wallis analysis test. Scale bar, 5 μm. (E) Western blot analysis of logarithmically and post-logarithmically growing cultures of the strains indicated expressing Pex11-GFP and analyzed with western blotting using monoclonal anti-GFP and anti-Pgk1. The data underlying the graphs shown in the figure can be found in S1 Data. (TIF) [file pbio.3002567.s004.tif]
