# Supplementary material for: Noncanonical and reversible cysteine ubiquitination prevents the overubiquitination of PEX5 at the peroxisomal membrane
Source: PLoS Biol. 2024 Mar 12;22(3):e3002567. doi: 10.1371/journal.pbio.3002567 (PMC10959387; doi:10.1371/journal.pbio.3002567)
Supplement: S1 File — (DOCX) [file pbio.3002567.s005.docx]

**Supporting Information Text**

**S1 Text. DTM-embedded PEX5 is still not oligo/polyubiquitinated in the absence of GSH and cytosolic proteins**

To assess if oligo/polyubiquitinated PEX5 species are particularly susceptible to GSH or to some other component present in the cytosolic fraction, a two-step *in vitro* assay was performed. Briefly, radiolabeled PEX5 or PEX5(C11K) were incubated with a primed PNS in the presence of ADPNP to accumulate monoubiquitinated PEX5 species at the DTM. Organelles were then isolated, resuspended in buffer lacking GSH, and incubated in the presence of ADPNP and pre-charged E1/E2. As shown in S1 Fig, peroxisomal PEX5 was still not oligo/polyubiquitinated (lanes 3-6) whereas oligo/polyubiquitinated PEX5(C11K) species were easily detected (lanes 9-12).

**S2 Text. DTM-embedded monoubiquitinated PEX5(C11K) and soluble monoubiquitinated wild-type PEX5 are not deubiquitinated by the E2**

E2-mediated deubiquitination assays were performed exactly as described for Fig 5B (see main text and Materials and Methods) but using radiolabeled PEX5(C11K) instead. As expected, monoubiquitinated PEX5(C11K) was not deubiquitinated in the presence of active E2D3 (S2A Fig, lanes 2-6).

To assess if cytosolic/soluble monoubiquitinated PEX5 is deubiquitinated by the E2, soluble/cytosolic Ub-PEX5 prepared as described in Materials and Methods was diluted 10-fold in buffer containing either 2 µM of uncharged recombinant E2D3 or E2D3(C85A) and 20 mM EDTA to stop *de novo* ubiquitination. As shown in S2 Fig, no deubiquitination of Ub-PEX5 was observed, indicating that only DTM-embedded Ub-PEX5 is deubiquitinated by E2D3.

**S3 Text. Yeast cells expressing PEX5C6K do not display an increase in pexophagy**

To monitor pexophagy in *pex5*∆ yeast cells expressing either WT Pex5 or *pex5C6K*, we co-expressed Pex11-GFP in cells by growing them on oleate medium for 16 h to induce peroxisome proliferation and high expression of Pex11-GFP. Subsequently, cells were transferred to a glucose medium lacking a nitrogen source. These conditions induce a strong pexophagy response [1]. Accumulation of the relatively protease-resistant GFP is indicative of vacuolar breakdown of Pex11-GFP [2]. Under these pexophagy-inducing conditions, *pex1∆* cells have increased breakdown of Pex11-GFP compared to WT PEX5 and *pex5C6K* cells (S3A Fig). Both WT Pex5 and *pex5C6K* mutant cells expressed Pex11-GFP to comparable levels and the GFP breakdown products started to accumulate after 2 h of starvation at comparable levels, and this trend continued. No significant difference in pexophagy after 6 h nitrogen starvation was detected using western blot analysis (S3B Fig). Even when cells are grown on oleate for 16 h, a low level of pexophagy was observed in *pex1∆* cells which agrees with previous work ([3]; see also S3A Fig). Live cell imaging of WT Pex5 and *pex5C6K* cells expressing Pex11-GFP grown on oleate and after 6 h starvation, revealed no obvious differences in peroxisome number (S3C Fig and S3D Fig). In contrast, in *pex1∆* cells, peroxisomal membrane structures containing Pex11-GFP were strongly reduced even during conditions of peroxisome proliferation (S3C Fig and S3D Fig). In agreement with previous observations, *pex1∆* cells induce pexophagy in post-logarithmically growing cultures resulting in low levels of Pex11-GFP ([3]; see also S3E Fig). *pex5C6K* mutant cells were indistinguishable from WT cells under these growth conditions (S3E Fig). We conclude *pex5C6K* cells are not noticeably affected in our pexophagy assays in *S. cerevisiae.*

**Supporting Information Text References**

1. Hutchins MU, Veenhuis M, Klionsky DJ. Peroxisome degradation in Saccharomyces cerevisiae is dependent on machinery of macroautophagy and the Cvt pathway. J Cell Sci. 1999;112: 4079–4087. doi:10.1242/jcs.112.22.4079

2. Motley AM, Nuttall JM, Hettema EH. Pex3-anchored Atg36 tags peroxisomes for degradation in Saccharomyces cerevisiae. EMBO J. 2012;31: 2852–68. doi:10.1038/emboj.2012.151

3. Nuttall JM, Motley AM, Hettema EH. Deficiency of the exportomer components Pex1, Pex6, and Pex15 causes enhanced pexophagy in Saccharomyces cerevisiae. Autophagy. 2014;10: 835–845. doi:10.4161/auto.28259
