# Supplementary material for: Noncanonical and reversible cysteine ubiquitination prevents the overubiquitination of PEX5 at the peroxisomal membrane
Source: PLoS Biol. 2024 Mar 12;22(3):e3002567. doi: 10.1371/journal.pbio.3002567 (PMC10959387; doi:10.1371/journal.pbio.3002567)

Figure 1 Raw Images

Left panel

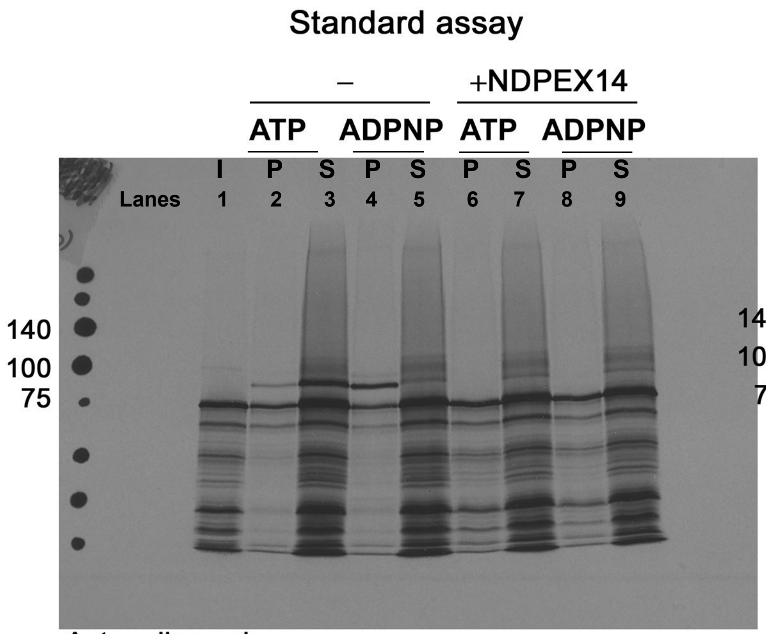

Autoradiography

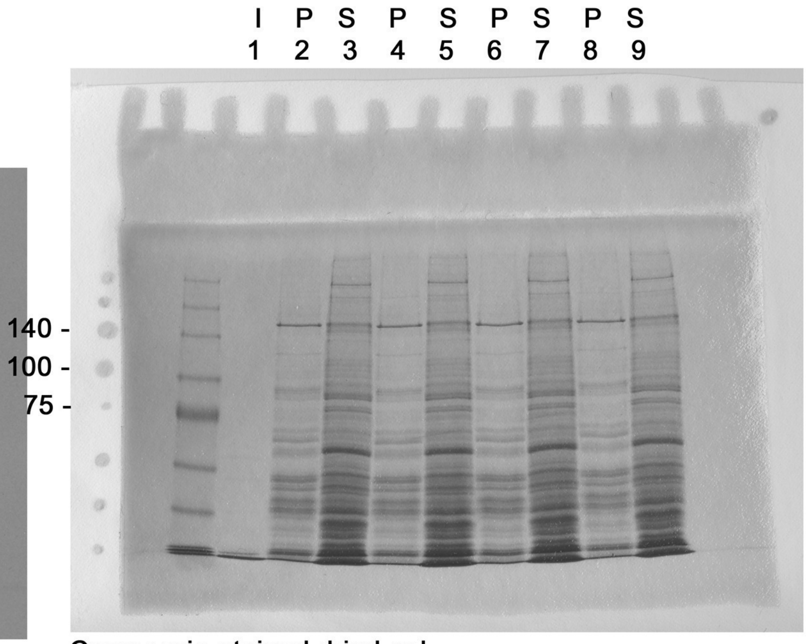

Coomassie-stained dried gel

Right panel

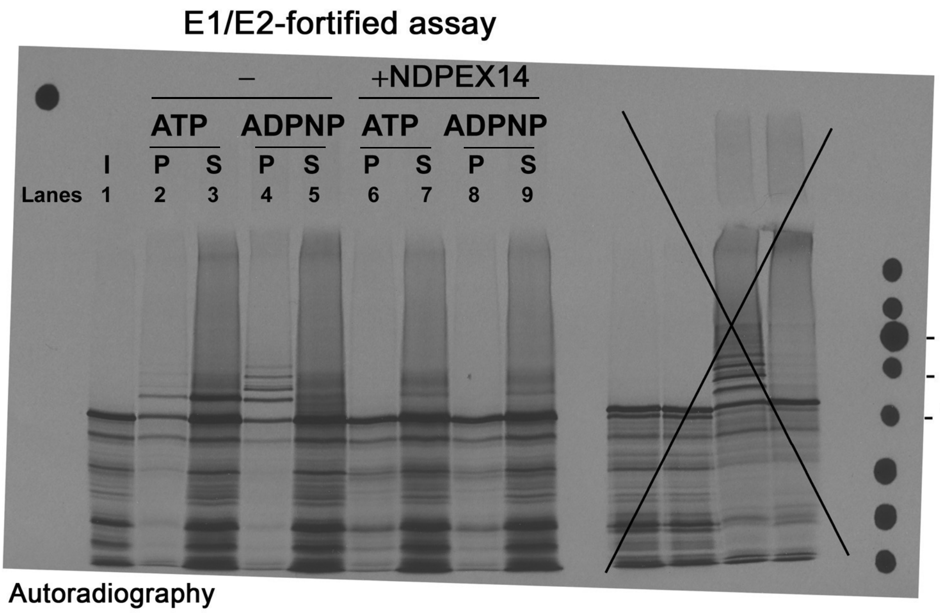

Autoradiography

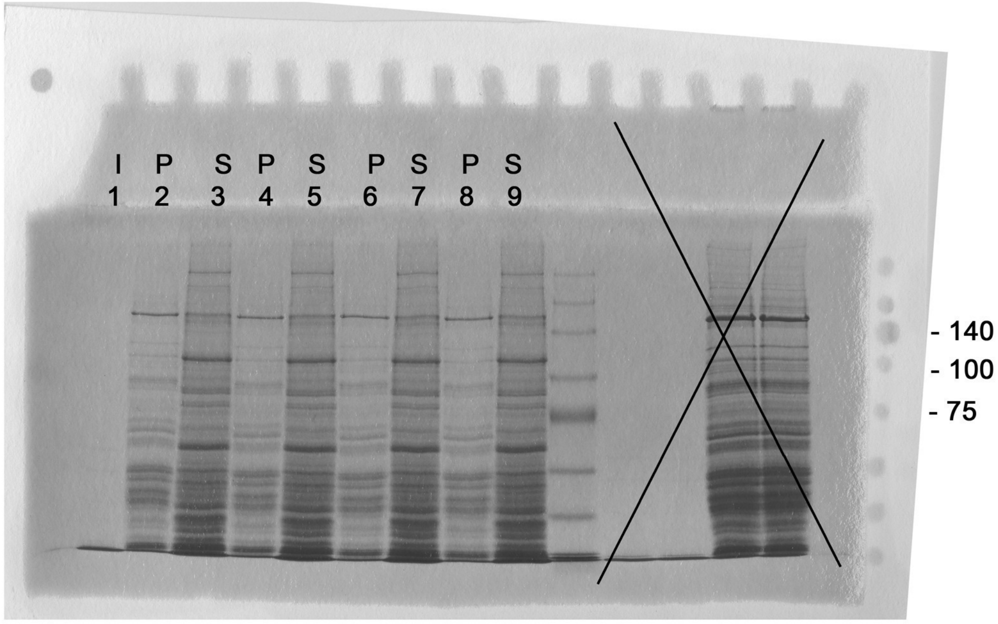

Coomassie-stained dried gel

Figure 2 Raw Images

Panel 2A

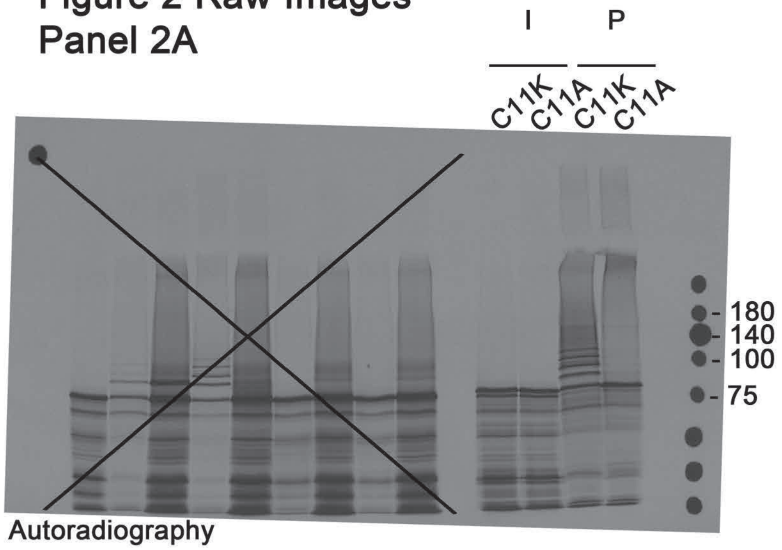

Autoradiography

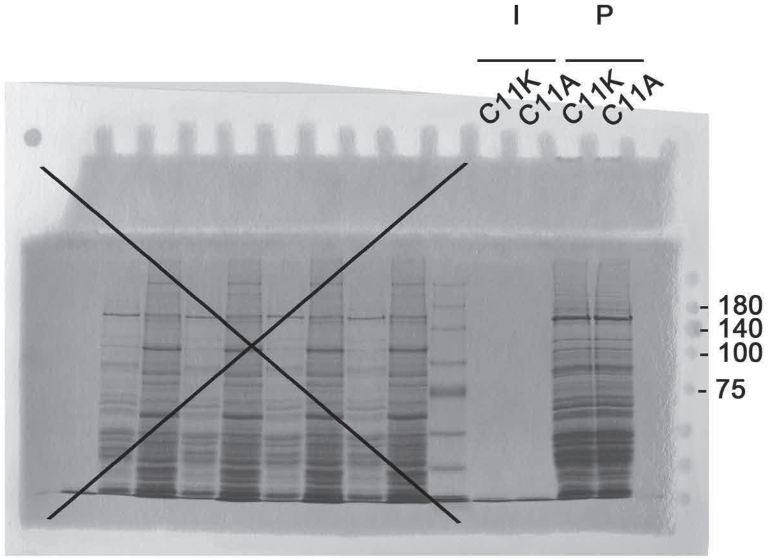

Coomassie-stained dried gel

Panel 2B

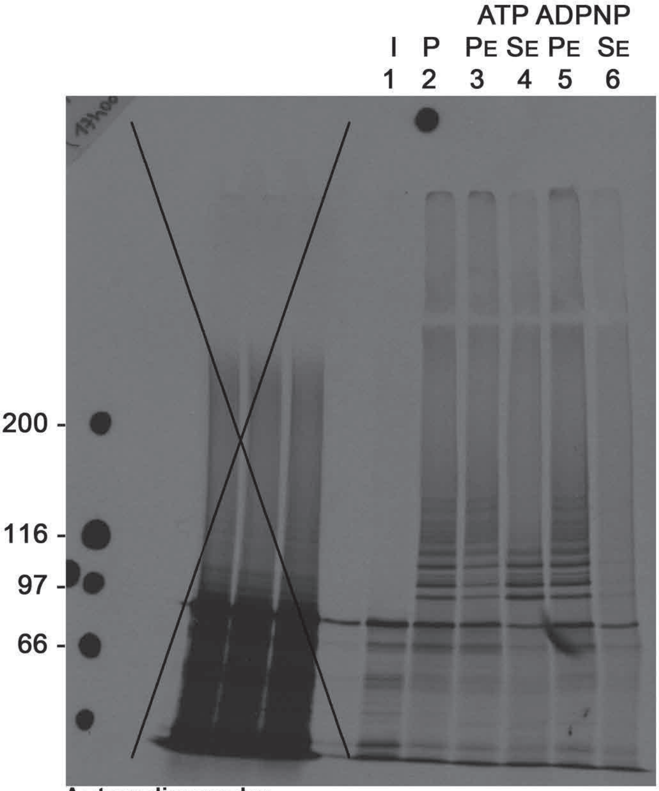

Autoradiography

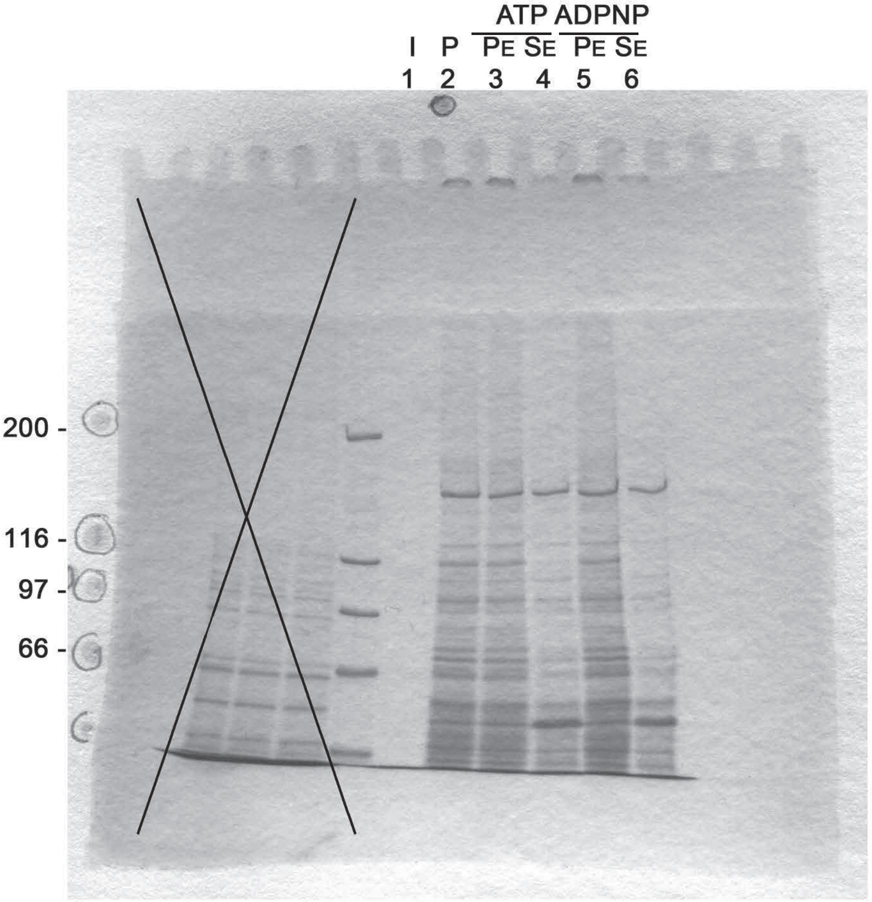

Coomassie-stained dried gel

Panel 2C

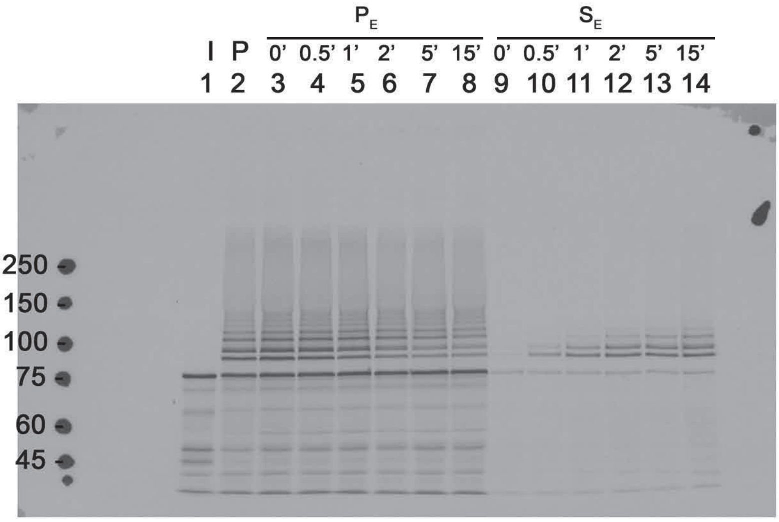

Autoradiography

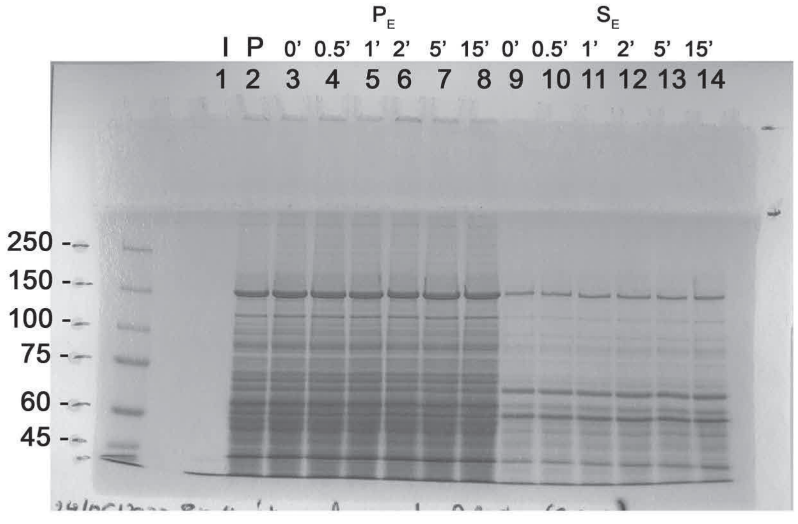

Coomassie-stained dried gel

Figure 3 Raw Image
Panel 3

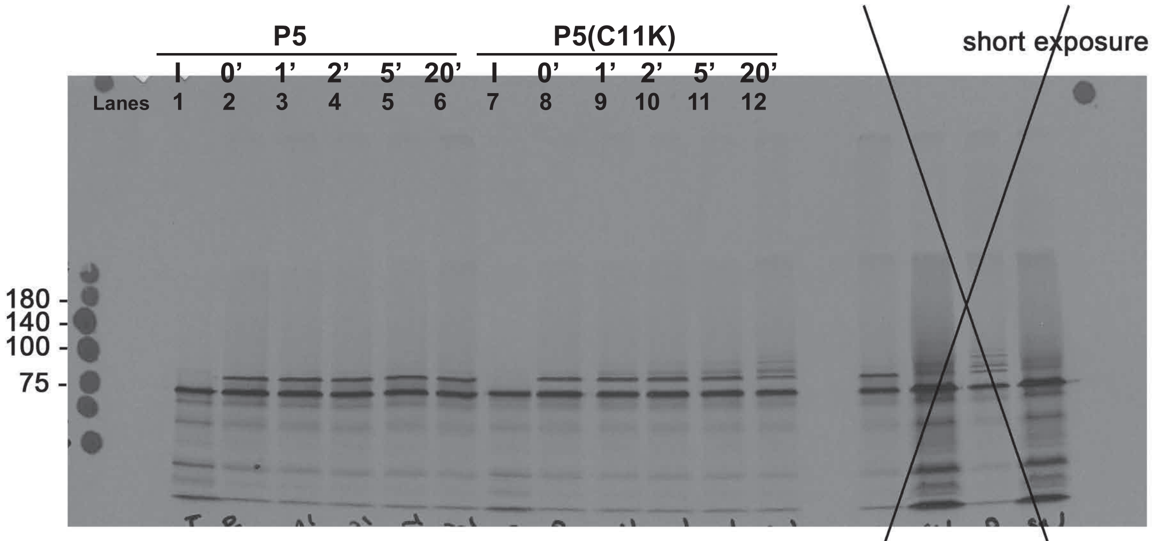

Autoradiography

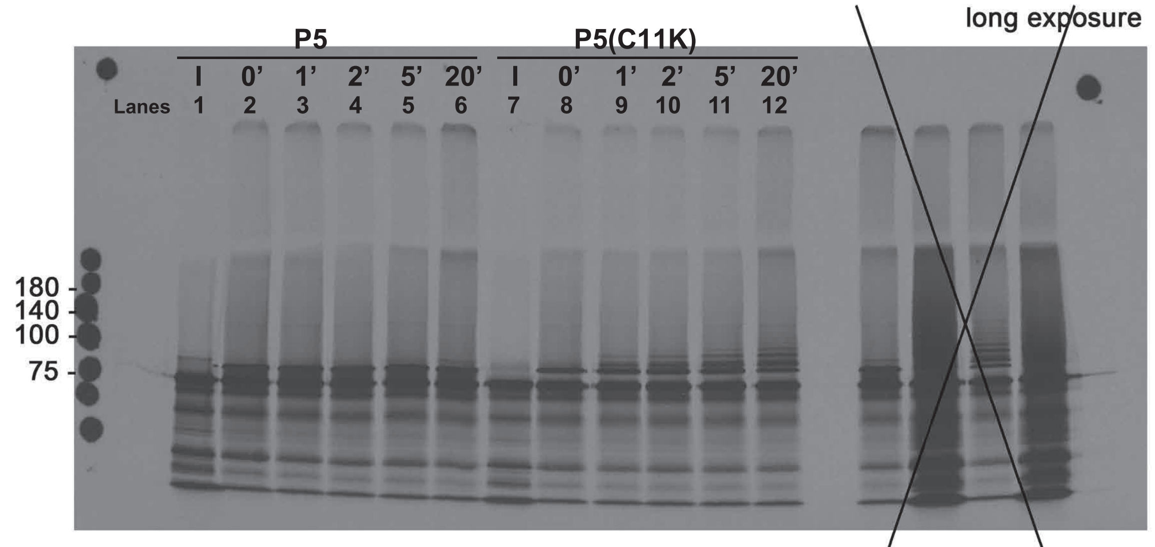

Autoradiography

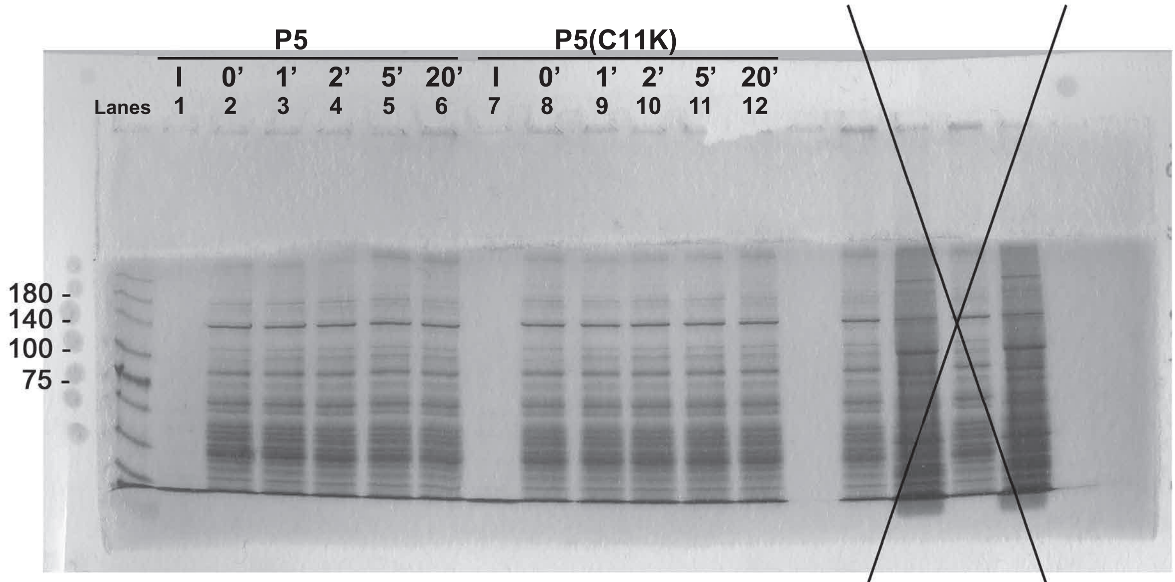

Coomassie-stained dried gel

Figure 4 Raw Image
Panel 4B

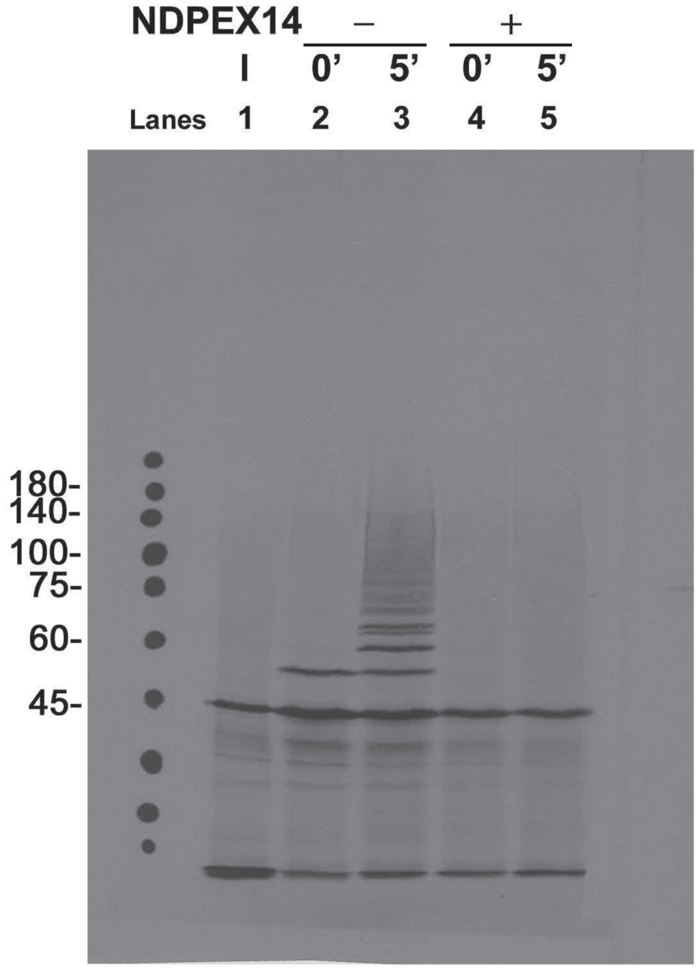

Autoradiography

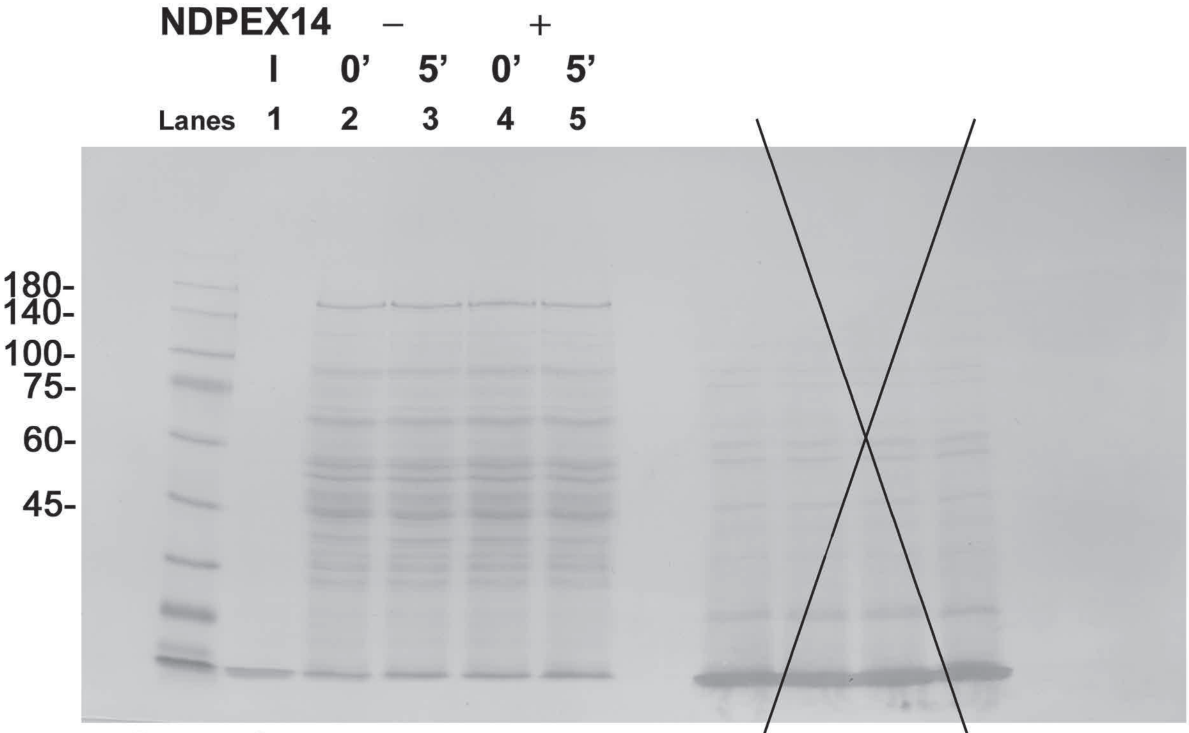

Ponceau S

Figure 5 Raw Image

Panel 5A

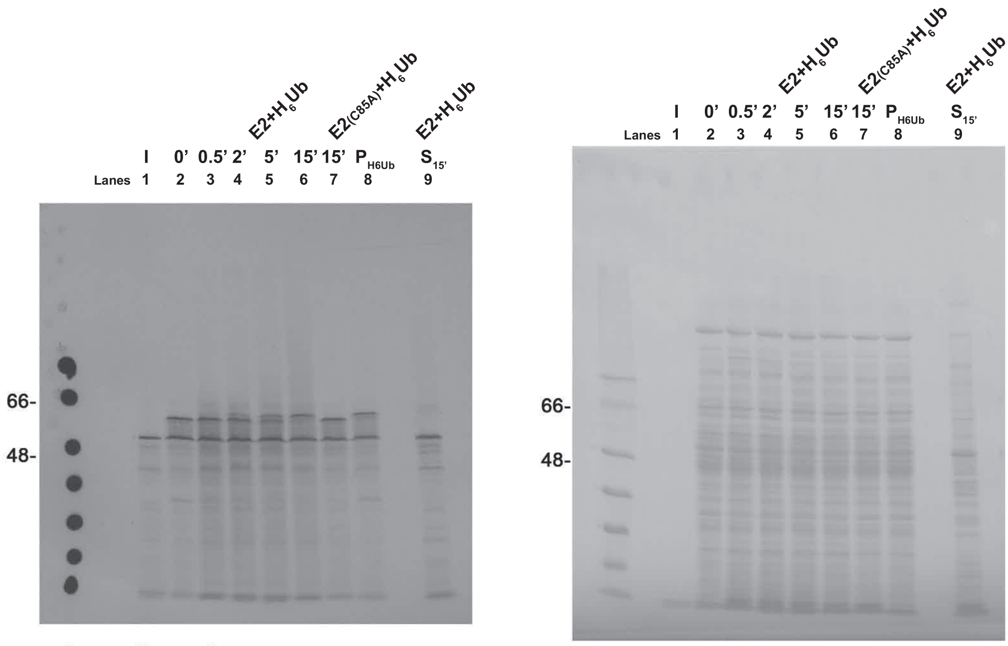

Autoradiography

Ponceau S

Panel 5B

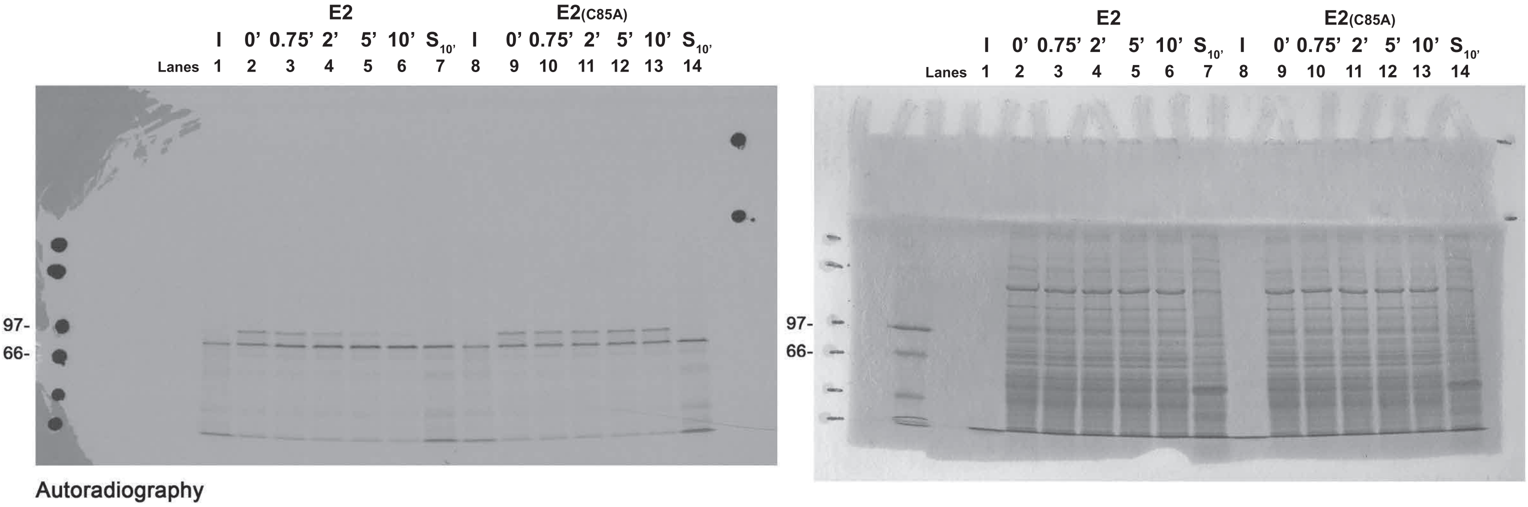

Autoradiography

Coomassie-stained dried gel

Panel 5C

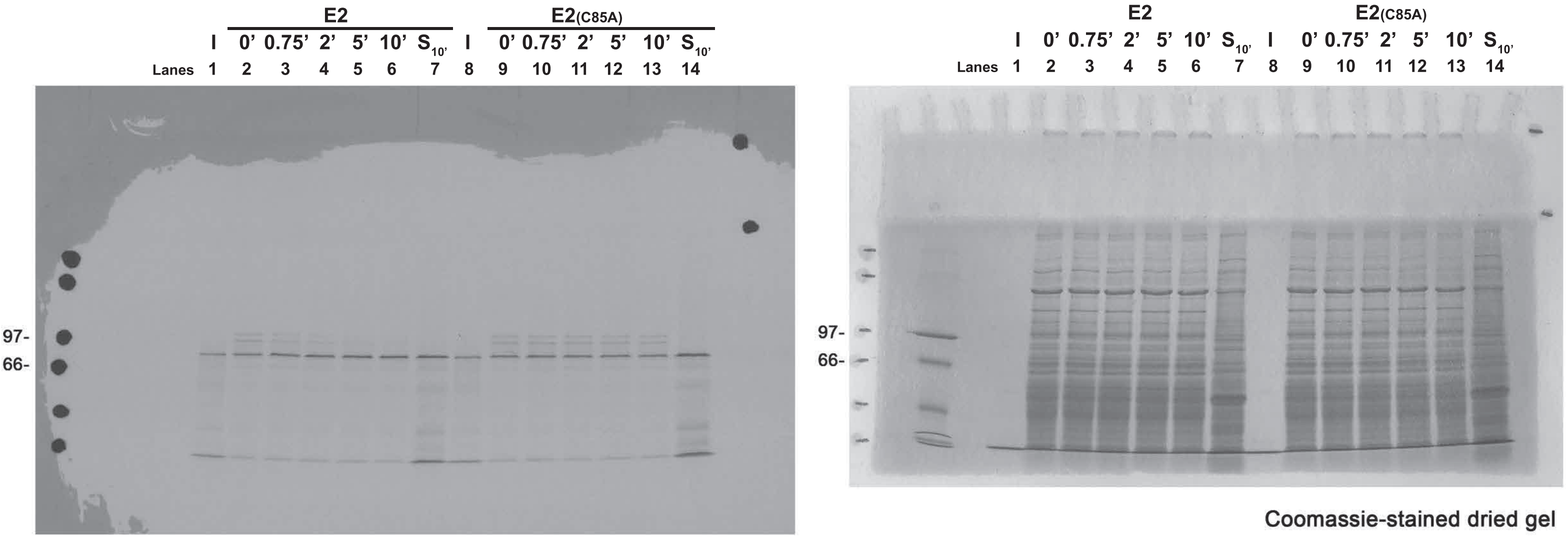

Coomassie-stained dried gel

Autoradiography

Figure S1 Raw Image

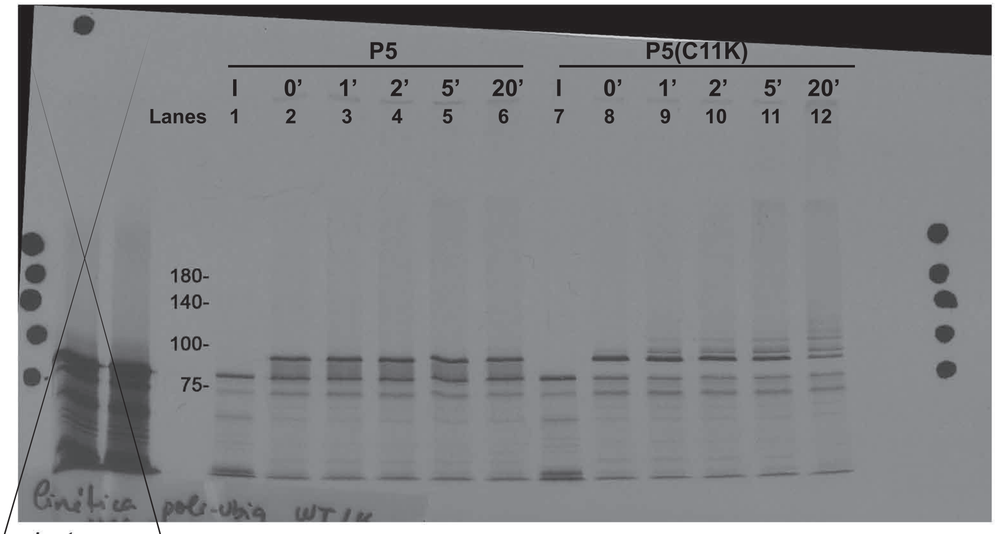

short exposure

Autoradiography

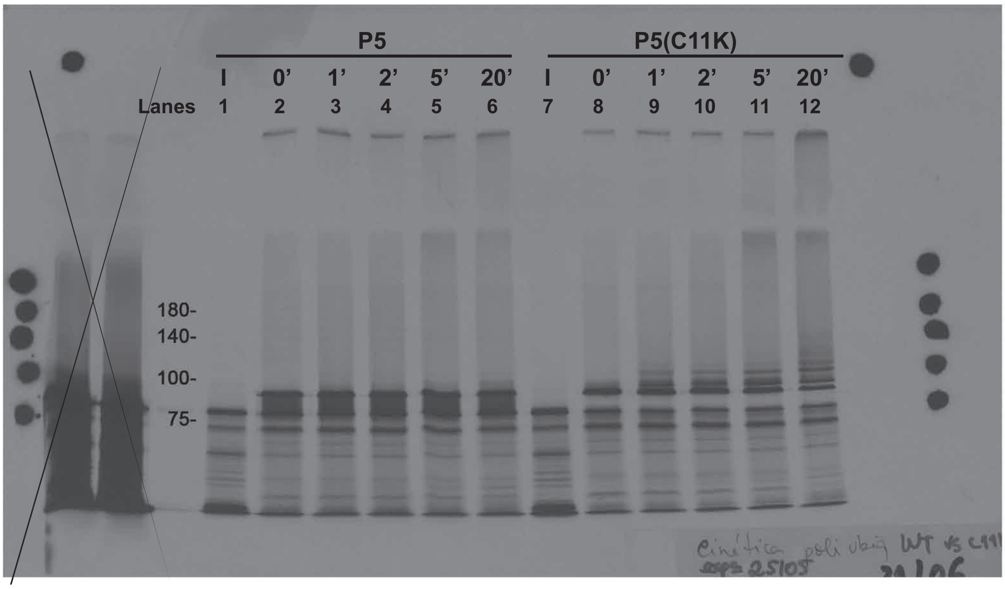

long exposure

Autoradiography

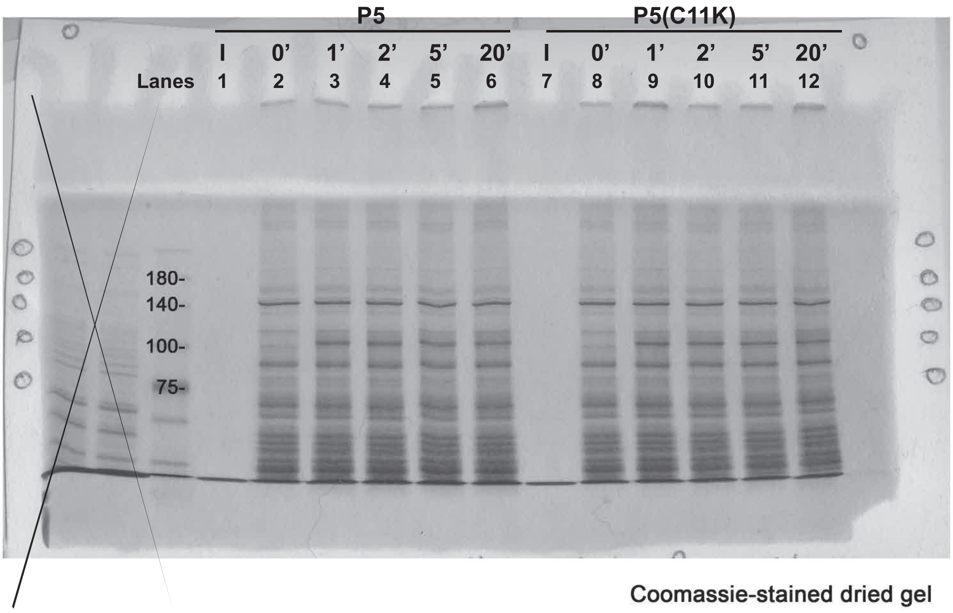

Coomassie-stained dried gel

Figure S2 Raw Image

Panel A

E2 E2(C85A)

I 0' 0.75' 2' 5' 10' $S_{10'}$ I 0' 0.75' 2' 5' 10' $S_{10'}$

Lanes 1 2 3 4 5 6 7 8 9 10 11 12 13 14

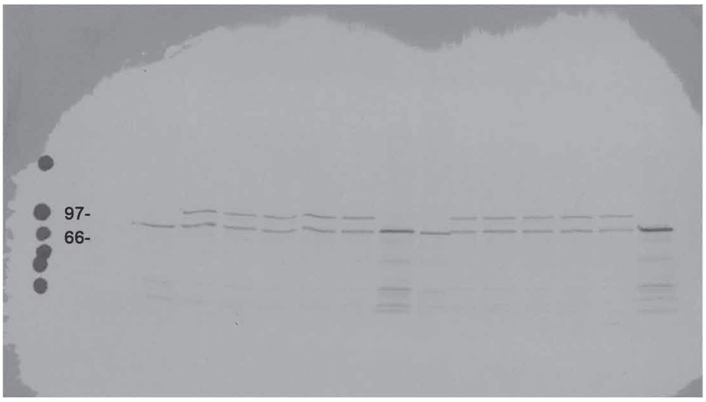

Autoradiography

E2 E2(C85A)

I 0' 0.75' 2' 5' 10' $S_{10'}$ I 0' 0.75' 2' 5' 10' $S_{10'}$

Lanes 1 2 3 4 5 6 7 8 9 10 11 12 13 14

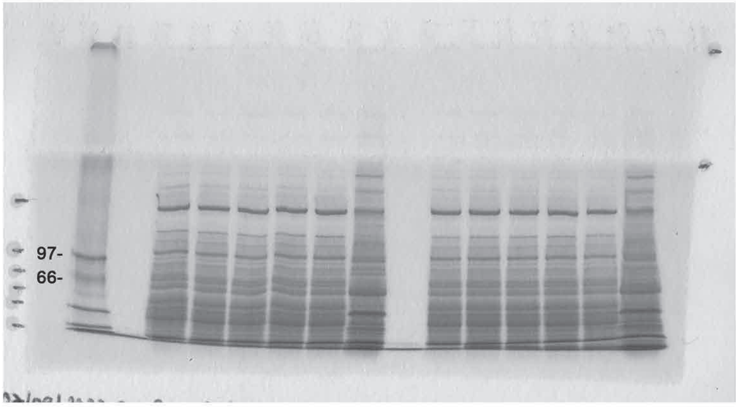

Coomassie-stained dried gel

Panel B

S_E + E2 S_E + E2(C85A)

I P P_E 0' 2' 10' I P P_E 0' 2' 10'

Lanes 1 2 3 4 5 6 7 8 9 10 11 12

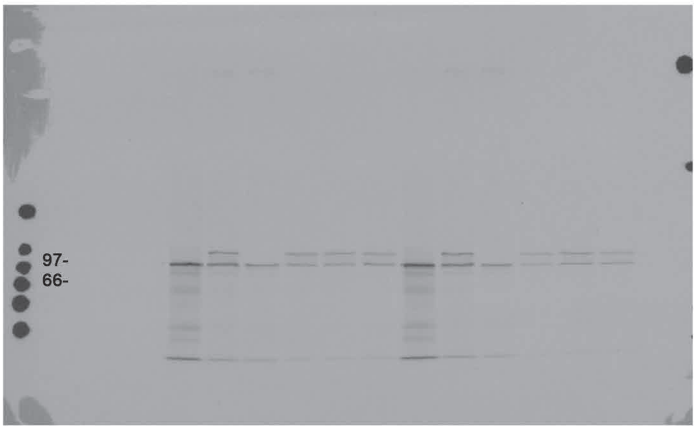

Autoradiography

S_E + E2 S_E + E2(C85A)

I P P_E 0' 2' 10' I P P_E 0' 2' 10'

Lanes 1 2 3 4 5 6 7 8 9 10 11 12

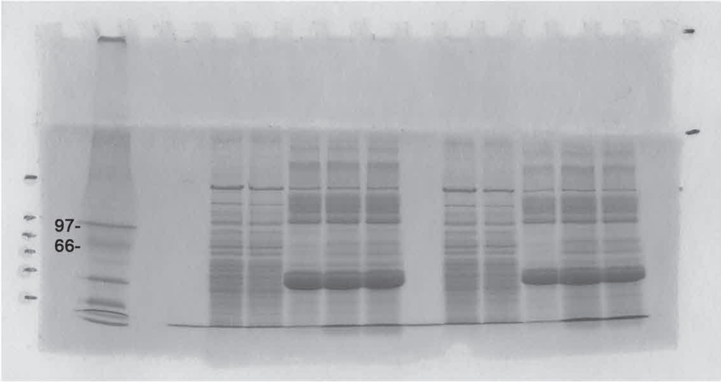

Coomassie-stained dried gel

Figure S3 Raw Images

Panel A

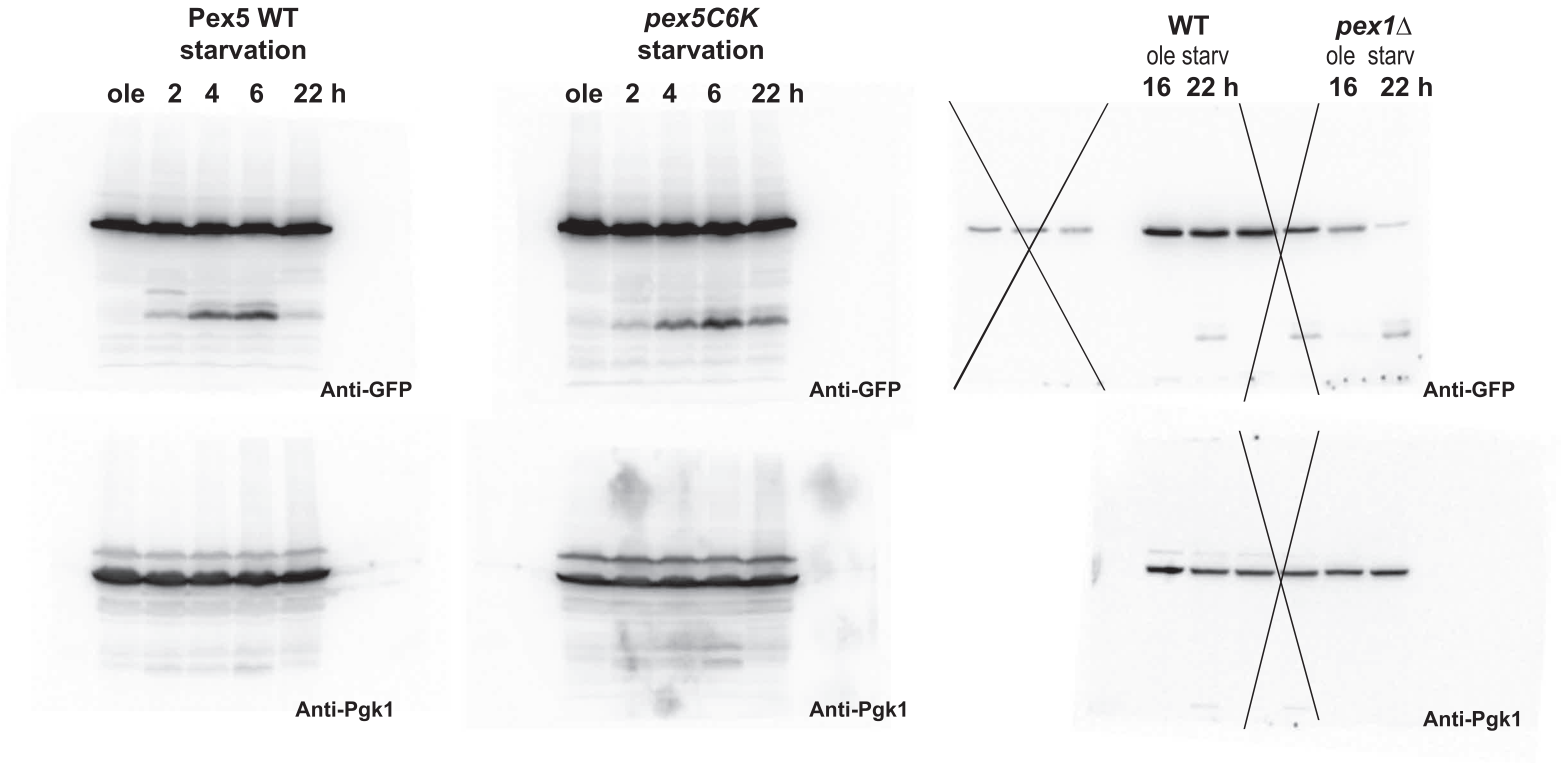

Panel E

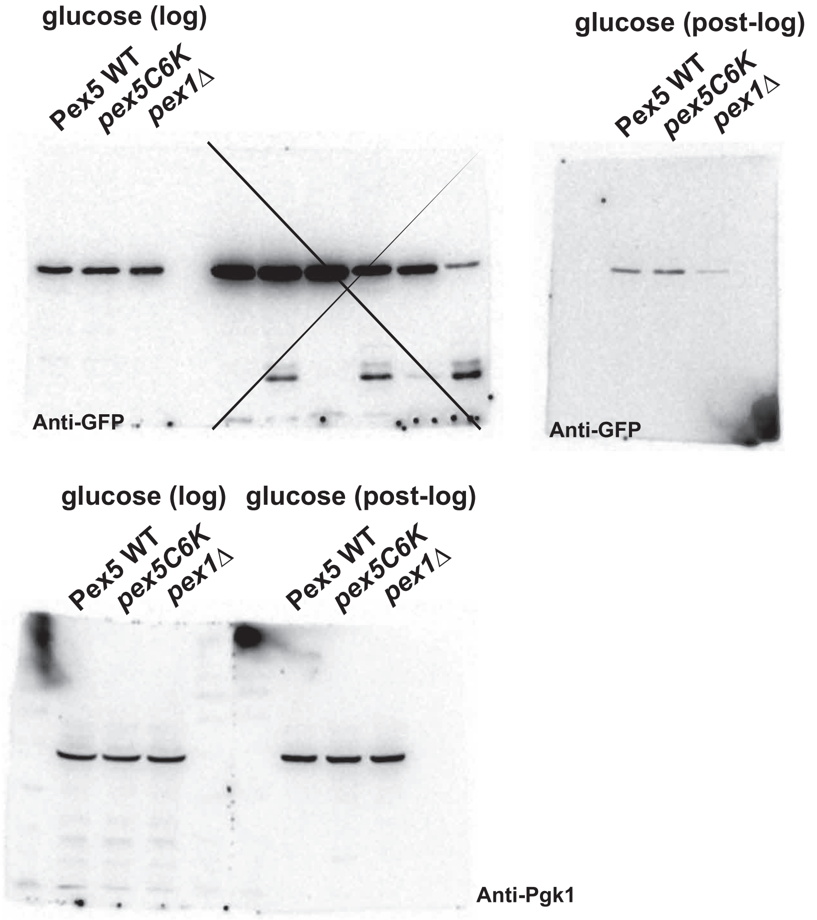

Supplement: S1 Raw Images — The molecular weights of the ladder in kiloDaltons (kDa) are shown for each blot. (PDF) [file pbio.3002567.s006.pdf]
