# Supplementary material for: Noncanonical and reversible cysteine ubiquitination prevents the overubiquitination of PEX5 at the peroxisomal membrane
Source: PLoS Biol. 2024 Mar 12;22(3):e3002567. doi: 10.1371/journal.pbio.3002567 (PMC10959387; doi:10.1371/journal.pbio.3002567)
Supplement: S1 Data — (PDF) [file pbio.3002567.s007.pdf]

Data set of Figure 3

Densitometric analysis of the conversion of monoubiquitinated PEX5(C11K) to oligo/polyubiquitinated species over time.

	Protein/lane*	Correction factor	Ub-P5(C11K)	Normalized Ub-P5(C11K)#	% Ub-P5(C11K) in organelles
**Time (min)**	**Experiment 1 (PNS 1)**				
0	44810	1.00	2350	2350	100.00
1	43128	0.96	2208	2294	97.62
2	47354	1.06	2292	2168	92.26
5	49454	1.10	2044	1852	78.78
10	-	-	-	-	-
20	46944	1.05	938	895	38.10
**Time (min)**	**Experiment 2 (PNS 2)**				
0	34800	1.00	2550	2550	100.00
1	37117	1.07	2379	2230	87.47
2	38338	1.10	2280	2069	81.16
5	37920	1.09	1720	1578	61.90
10	-	-	-	-	-
20	44013	1.26	792	626	24.54
**Time (min)**	**Experiment 3 (PNS 2)**				
0	56616	1.00	7872	7872	100.00
1	64048	1.13	7260	6418	81.52
2	61754	1.09	7495	6871	87.28
5	62212	1.10	6842	6227	79.10
10	60034	1.06	5468	5157	65.50
20	58075	1.03	2618	2552	32.42
**Time (min)**	**Experiment 4 (PNS 2)**				
0	55010	1.00	6054	6054	100.00
1	-	-	-	-	-
2	57440	1.04	5389	5161	85.25
5	56025	1.02	4650	4566	75.42
10	58355	1.06	3835	3615	59.71
20	58480	1.06	1871	1760	29.07

* Protein/lane values were obtained by densitometric analysis of coomassie-blue stained gels. All lanes were normalized to the amount present in the "Time 0" sample.

"Ub-P5(C11K)" monoubiquitinated radiolabeled PEX5(C11K).

The amount of radiolabeled Ub-P5(C11K) in each lane was normalized to the amount of protein of in the respective lane.

"-" non-determined.

Raw Data used for quantifications

Experiment 1

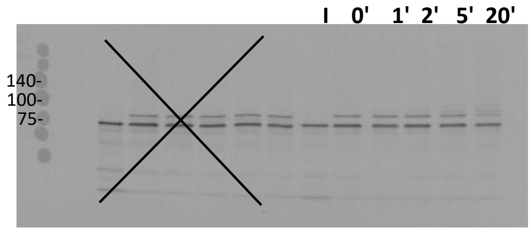

Autoradiography

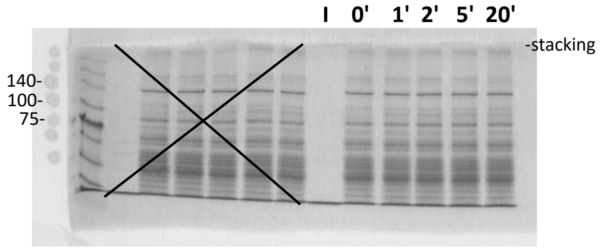

Coomassie-stained dried gel

Experiment 2

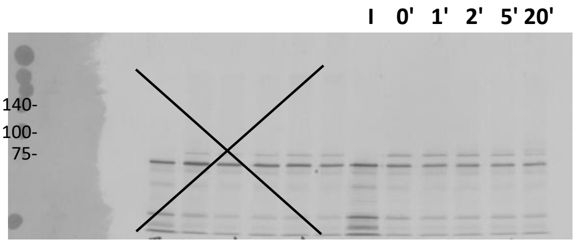

Autoradiography

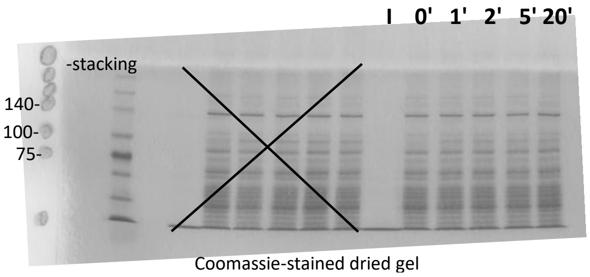

Coomassie-stained dried gel

Experiment 3

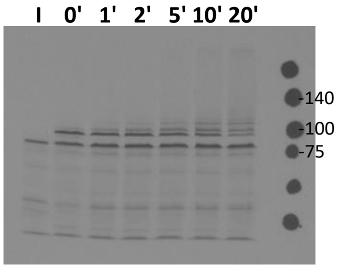

Autoradiography

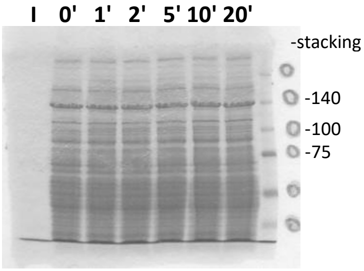

Coomassie-stained dried gel

Experiment 4

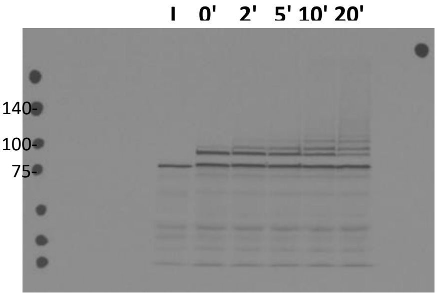

Autoradiography

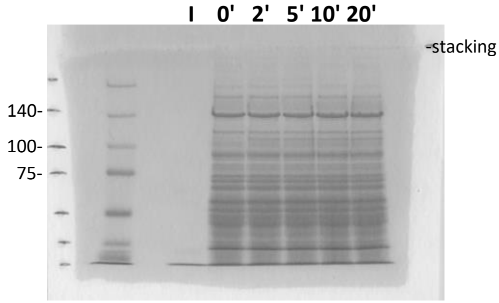

Coomassie-stained dried gel

Data set of Figure 5B

Densitometric analysis of the E2D3-mediated deubiquitination of monoubiquitinated PEX5 over time.

	Protein/lane*	Correction factor	Ub-P5	Normalized Ub-P5#	% Ub-P5 in organelles
**Time (min)**			**Experiment 1**		
0	42507	1.00	3197	3197	100.00
0.75	42800	1.01	2019	2005	62.72
2	40512	0.95	1349	1416	44.28
5	42707	1.00	830	826	25.83
10	42459	1.00	262	262	8.19
**Time (min)**			**Experiment 2**		
0	33542	1.00	3365	3365	100.00
0.75	35959	1.07	2224	2074	61.63
2	35342	1.05	1273	1208	35.89
5	36708	1.09	676	618	18.35
10	33575	1.00	318	318	9.44
**Time (min)**			**Experiment 3**		
0	44507	1.00	1559	1559	100.00
0.75	41789	0.94	1172	1249	80.06
2	39080	0.88	556	633	40.59
5	40860	0.92	288	314	20.13
10	40542	0.91	234	257	16.48

* Protein/lane values were obtained by densitometric analysis of coomassie-blue stained gels. All lanes were normalized to the amount present in the "Time 0" sample.

"Ub-P5" monoubiquitinated radiolabeled PEX5.

The amount of radiolabeled Ub-P5 in each lane was normalized to the amount of protein of in the respective lane.

Raw Data used for quantifications

Experiment 1

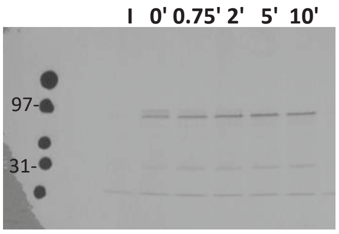

Autoradiography

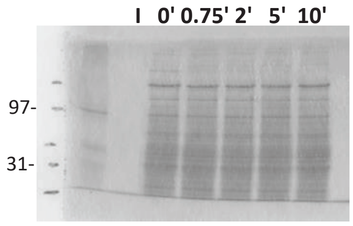

Coomassie-stained dried gel

Experiment 2

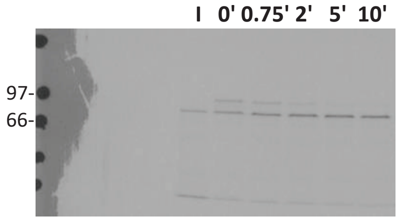

Autoradiography

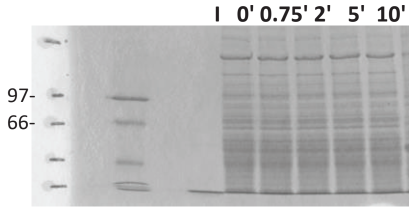

Coomassie-stained dried gel

Experiment 3

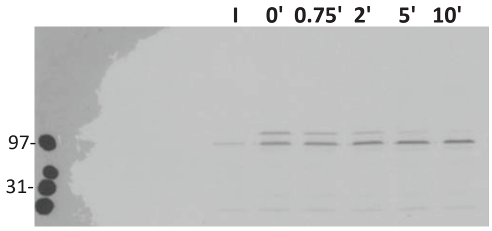

Autoradiography

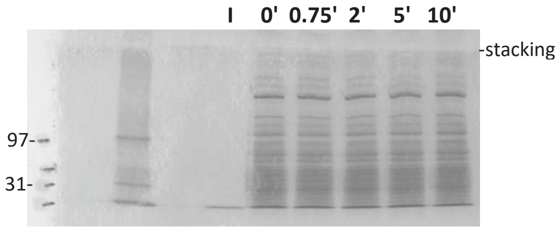

Coomassie-stained dried gel

Data S3B - PEX11-GFP/GFP

		replica's			
**Strains**		1st	2nd	3rd	4th
**Pex5 WT t=6**	Pex11-GFP	0.92626379	0.91754532	0.93454764	0.79826508
	GFP	0.07373621	0.08245468	0.06545236	0.20173492

**Pex5 C6K t=6**	Pex11-GFP	0.83333181	0.86145065	0.95497646	0.89826508
	GFP	0.16666819	0.13854935	0.04502354	0.27323035

Data set of Figure S3D - GFP puncta per cell

Oleate growth		
Pex5	Pex5C6K	*Δpex1*
6	2	2
11	0	1
6	9	3
8	9	3
4	3	2
12	0	3
12	9	4
7	10	2
6	12	2
5	12	2
8	12	0
15	10	2
2	6	1
9	11	0
6	6	0
4	15	0
12	7	2
7	14	0
10	18	1
8	0	2
9	10	2
13	12	1
9	10	4
3	8	3
3	9	8
10	4	0
9	5	0
9	4	0
0	3	0
3	8	0
15	7	0
6	10	1
5	3	1
4	5	1
12	0	1
8	6	4
14	9	7
6	6	0
13	5	0
0	7	4
11	3	0
13	6	3
3	10	1
8	8	4
3	9	4
1	9	1
2	12	3
4	11	0
15	8	0
3	9	0
4	14	0
13	4	0
4	20	0

Nitrogen Starvation		
Pex5	Pex5C6K	*Δpex1*
1	7	2
4	2	0
8	1	1
10	9	2
6	0	2
3	6	1
3	7	2
5	6	4
8	6	0
2	3	1
9	8	1
9	5	1
7	5	1
5	8	1
7	8	0
0	6	1
2	3	2
3	6	2
5	8	2
4	9	2
5	6	0
3	5	0
10	9	0
3	9	1
2	7	2
5	5	2
5	5	1
5	7	0
4	5	3
9	4	1
4	1	1
2	7	1
5	7	3
4	8	2
4	0	2
5	6	1
0	1	1
5	6	1
4	5	1
3	5	0
3	5	2
3	7	4
5	6	3
8	3	4
8	7	0
6	5	0
10	7	0
9	6	0
6	8	0
4	7	0
9	4	4
5	10	2
5	8	3

12	7	1
6	6	2
9	5	2
7	14	3
7	14	0
8	6	0
3	6	2
4	0	2
12	5	3
7	7	5
8	5	3
8	3	2
3	6	3
8	10	5
3	11	2
27	5	1
12	9	2
9	9	2
18	0	1
9	7	2
5	14	2
1	9	2
22	9	2
0	4	0
4	10	0
6	3	3
3	13	0
7	5	2
0	3	2
12	6	2
11	0	1
6	5	2
11	8	0
12	9	2
10	5	2
11	6	1
2	3	2
5	7	3
6	8	4
8	5	4
8	10	0
14	10	2
7	9	4
9	7	3
12	14	5
7	5	0
7	15	2

3	9	0
5	6	1
5	3	1
4	7	2
0	9	4
3	7	0
10	7	0
6	2	2
6	4	3
2	10	4
5	4	1
4	3	1
4	6	2
6	8	0
0	0	0
4	0	2
5	7	2
7	5	3
3	8	2
2	5	2
1	10	2
5	7	1
1	1	0
7	7	2
5	5	2
4	6	3
4	6	0
6	4	4
7	7	8
5	6	3
6	8	2
7	7	0
5	3	2
5	6	2
5	4	4
4	4	1
7	9	2
1	5	0
4	4	1
7	10	2
7	3	1
9	6	1
8	4	3
4	6	2
6	8	3
7	10	1
8	6	2
